# Supplementary material for: Structure of a RecT/Redβ family recombinase in complex with a duplex intermediate of DNA annealing
Source: Nat Commun. 2022 Dec 21;13:7855. doi: 10.1038/s41467-022-35572-z (PMC9772228; doi:10.1038/s41467-022-35572-z)
Supplement: Supplementary file 1 — Supplementary Information [file 41467_2022_35572_MOESM1_ESM.pdf]

Supplementary Information for

Structure of a RecT/Redβ family recombinase in complex with a duplex intermediate of DNA annealing

Brian J. Caldwell, Andrew S. Norris, Caroline F. Karbowski, Alyssa M. Wiegand, Vicki H. Wysocki and Charles E. Bell

This document includes:

Supplementary Figs. 1 to 17
Supplementary Tables 1 to 4
Caption for Supplementary Movie 1
SI References

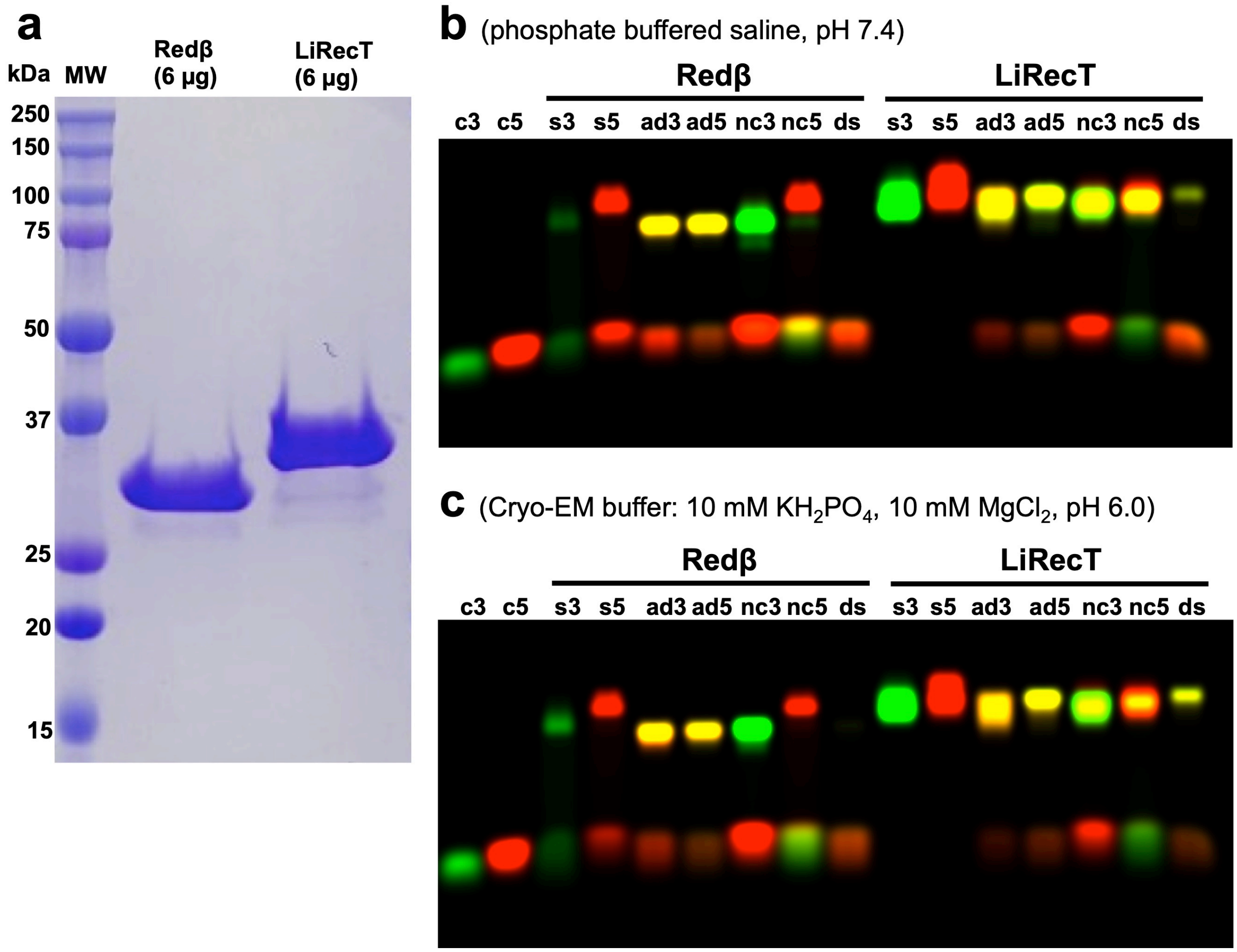

Supplementary Fig. 1 | Purification and DNA binding properties of LiRecT. **a**,12.5% SDS-PAGE gel stained with Coomassie Blue R250. **b,c,** Gel-based DNA annealing assay comparing the DNA binding activities of LiRecT with λ-Redβ in two different buffers: phosphate buffered saline (*b*), and the buffer used for cryo-EM grid preparation (*c*). Lanes contain the following complexes: c3 and c5: single 5'-labeled 50-mer oligo at 25 μM (nt) in the absence of protein; s3, s5: a single oligo at 25 μM with 5 μM protein; ad3, ad5: sequential addition of two complementary oligos, each at 25 μM, to 5 μM protein (with the indicated oligo added first); nc3, nc5: sequential addition of two non-complementary oligos to protein; ds: addition of two complementary pre-annealed oligos to protein. Notice that λ-Redβ binds weakly to ssDNA (as evidenced by the streaking in the s3 and s5 lanes), forms a distinct complex containing both oligos (yellow band) when two complementary oligos are added to the protein sequentially (ad3, ad5), does not form this complex when the two oligos are non-complementary (nc3, nc5), and forms no interaction with pre-formed dsDNA (ds). LiRecT exhibits similar behavior as λ-Redβ, but binds more tightly to ssDNA. Sequences of all oligonucleotides are described previously (1). The two gels in B and C were scanned with identical conditions. Source data are provided as a Source Data File. The experiments of panels a, b, and c were performed at least twice with similar results.

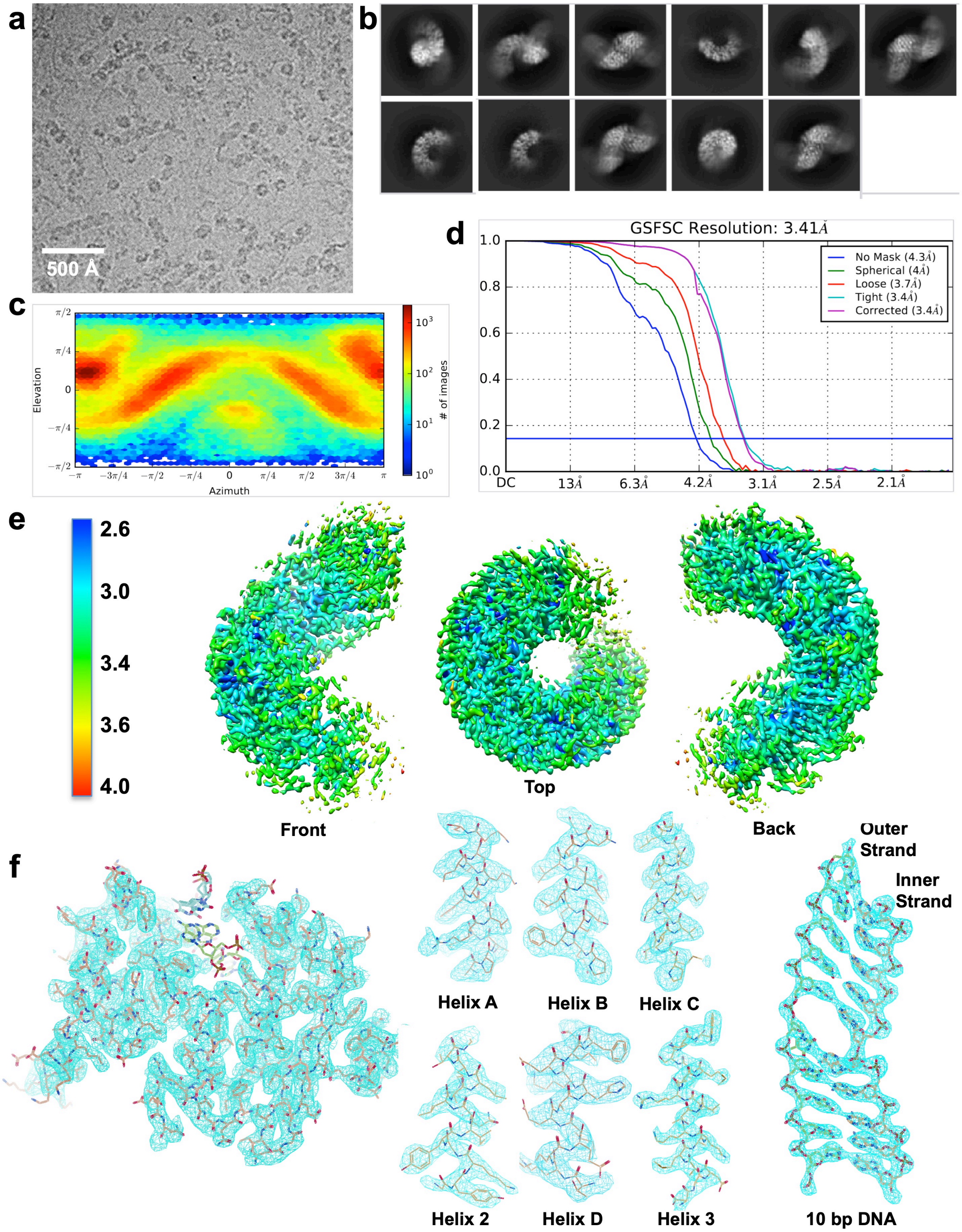

Supplementary Fig. 2 | Cryo-EM structure determination of LiRecT complex with 83-mer annealed duplex. a, Krios K3 image at 81,000x. The image is one of 2038 that gave similar results. **b**, example 2D class averages. **c**, angular distribution of particles used for final reconstruction. **d**, resolution estimate by gold-standard Fourier Shell Correlation (FSC). **e**, final 3D reconstruction colored by local resolution estimate. **f**, example regions of cryo-EM density for a central subunit of the filament (Chain I and associated DNA). In the leftmost figure the map is contoured around a complete LiRecT monomer looking down the DNA binding groove (the map is not contoured around the DNA in this figure). Figures were generated by cryoSPARC (2), UCSF Chimera (3), and COOT (4).

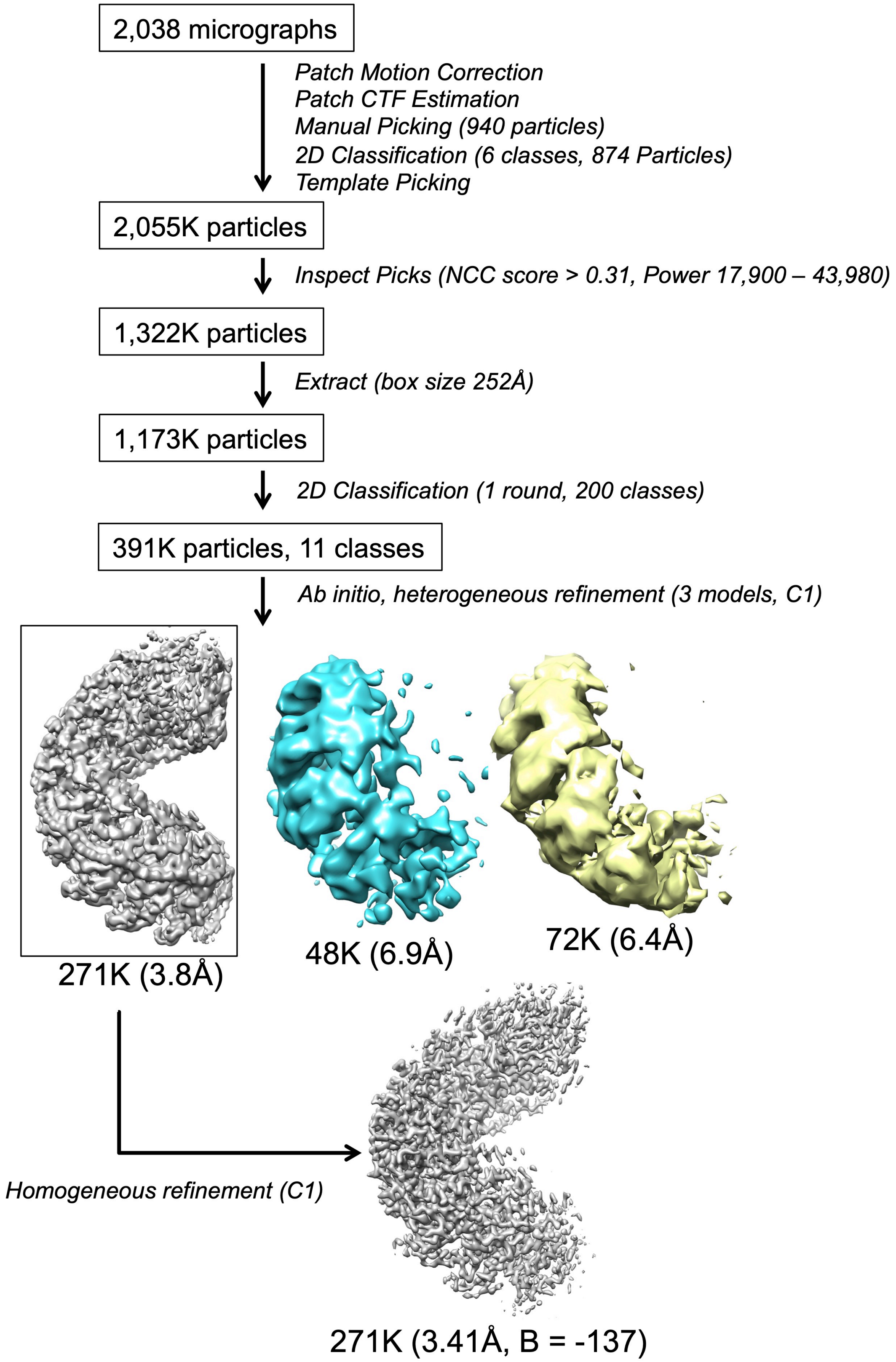

Supplementary Fig. 3 | CryoEM single particle workflow for complex of LiRecT with 83-mer annealed duplex. The analysis was performed in cryoSPARC (2) and the figures were drawn with UCSF Chimera (3).

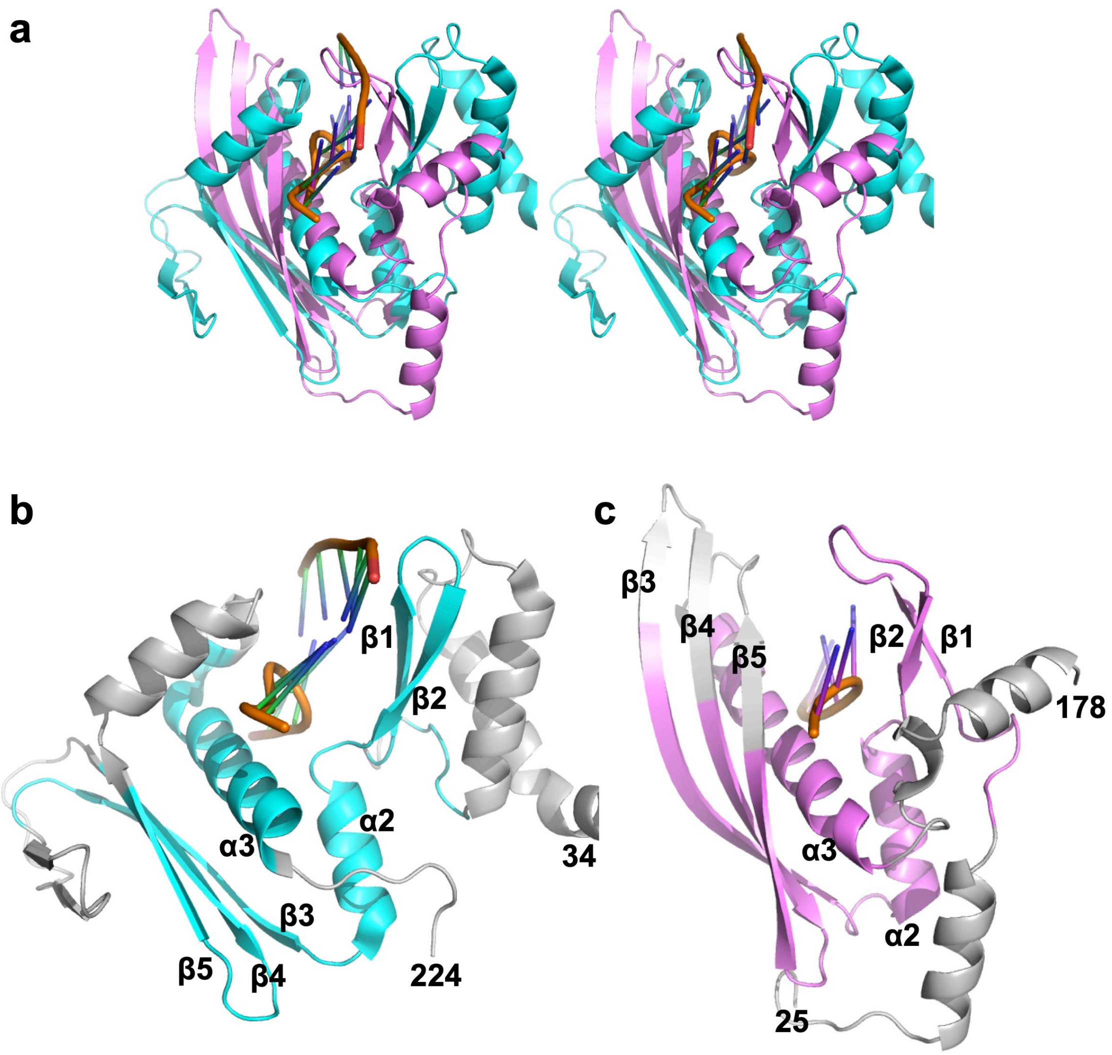

Supplementary Fig. 4 | Structural superposition of LiRecT and RAD52. a, Stereo view of a structural superposition of LiRecT and RAD52 output from the DALI server using the pairwise option (6). LiRecT is shown in cyan (chain J from PDB ID 7UB2) and RAD52 is shown in magenta (chain F from PDB ID 5xrz). The two structures superimpose to an rmsd of 5.5Å for 83 pairs of Cα atoms that share 14% sequence identity. The two strands of DNA bound to LiRecT (inner, outer) and the single strand of DNA bound to RAD52 (inner) are shown in the central groove. Notice that the two inner strands overlay closely. **b**, View of the LiRecT monomer with the segments that align with RAD52 as determined by DALI highlighted in cyan. These segments are residues 85-134 (β1, β2, α2, and β3), 160-176 (β4, β5) and 196-216 (α3). **c**, View of the RAD52 monomer with the segments that overlay with LiRecT as determined by DALI highlighted in magenta. These segments are residues 46-95 (β1, β2, α2, β3), 108-124 (β5, β5) and 137-158 (α3).

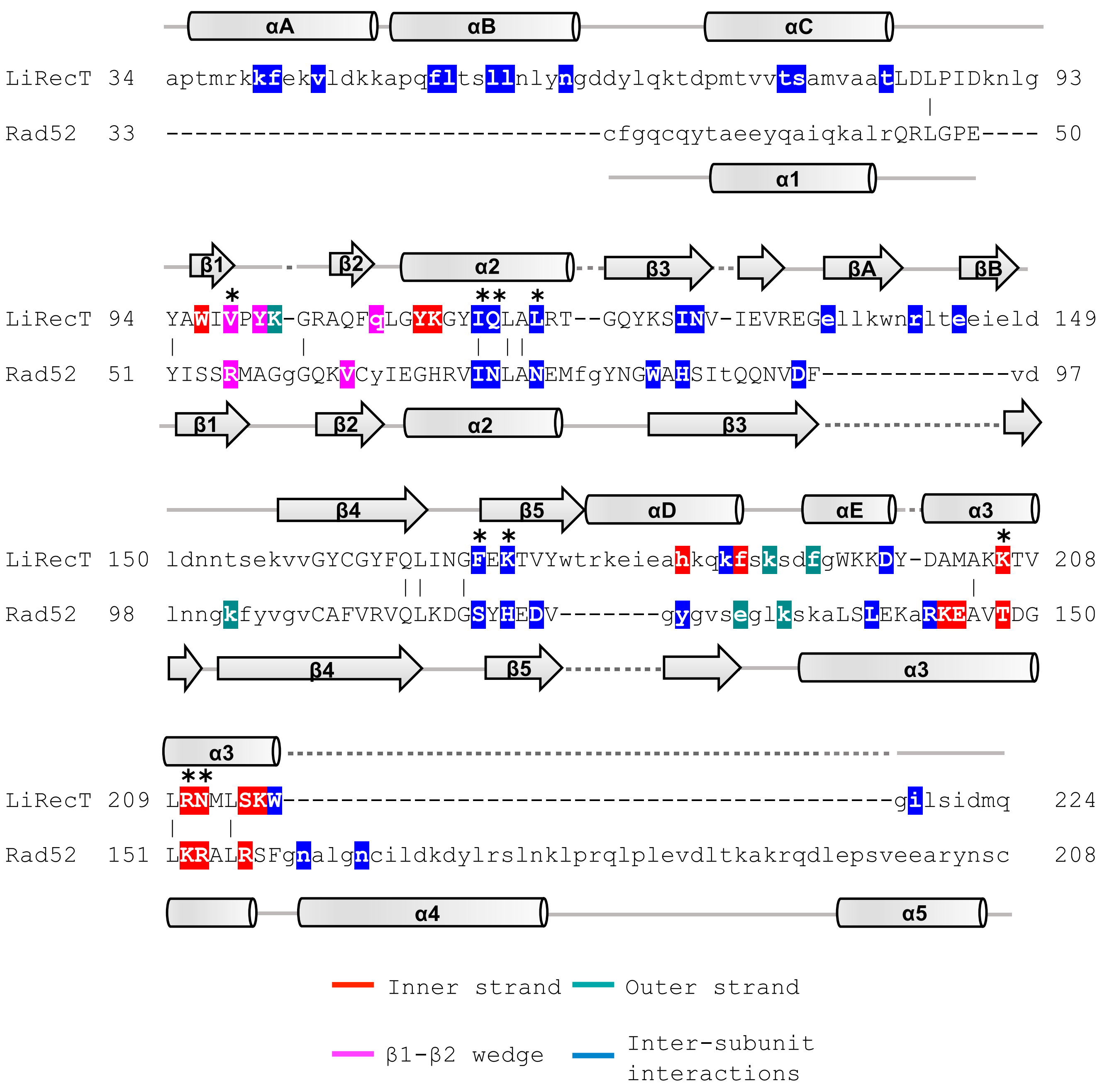

Supplementary Fig. 5 | Structure based sequence alignment of LiRecT and RAD52. Structurally equivalent residues in the alignment, based on a pairwise alignment using the DALI server (6), are shown in uppercase. Structurally non-equivalent residues (including insertions) are in lowercase. Amino acid identities are marked by vertical bars. The shading indicates amino acids of each protein that contact the inner strand (red), outer strand (teal), form the β1-β2 hairpin wedge (violet), or form inter-subunit interactions (blue). The ∗ symbols indicate key functional residues of LiRecT that are structurally conserved with RAD52 in the alignment.

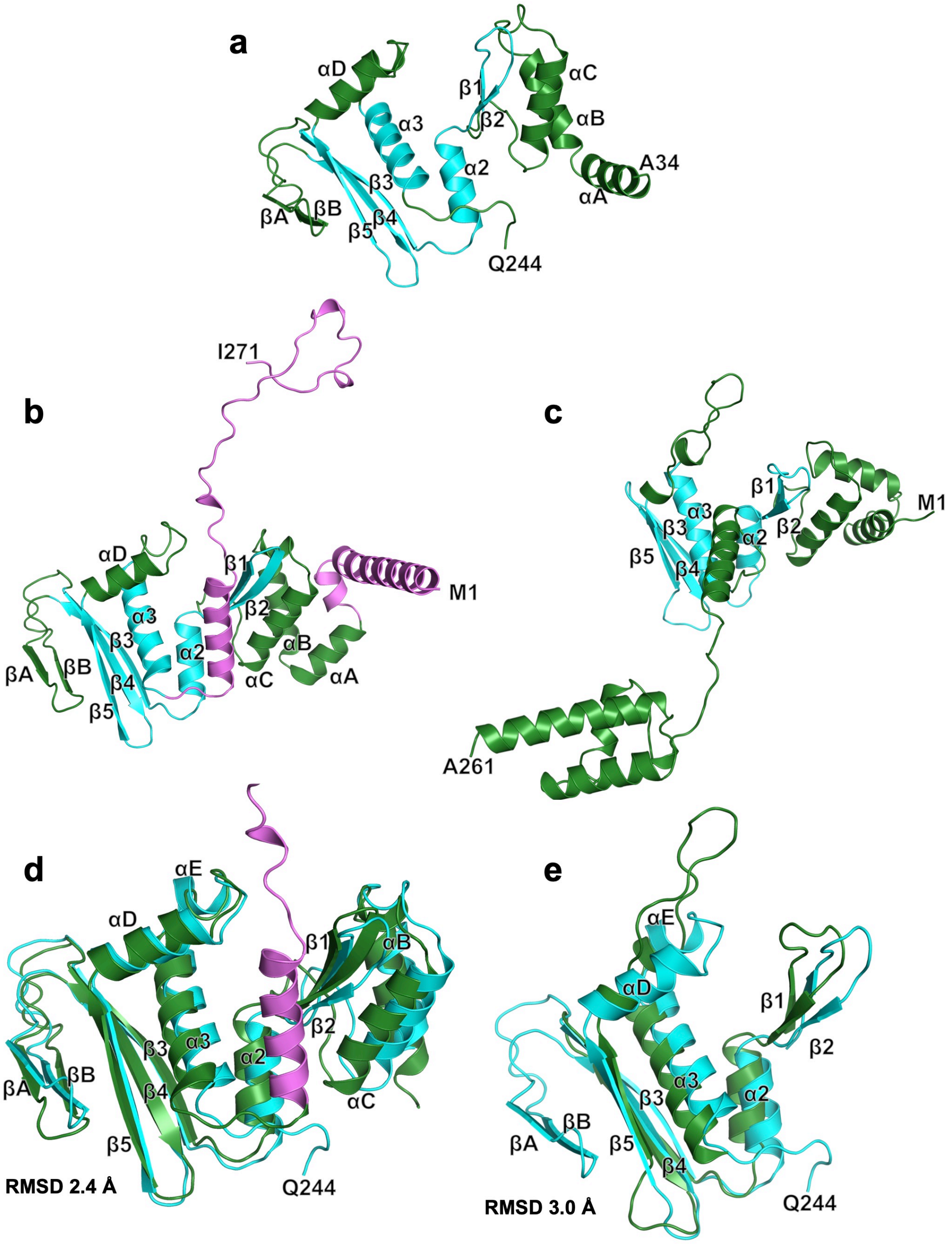

Supplementary Fig. 6 | Comparison of the LiRecT cryo-EM structure with the structures of LiRecT and λ-Redβ predicted by RoseTTAFold. **a**, Ribbon diagram of the LiRecT monomer from the cryo-EM structure. The core that is common to RAD52 is colored in cyan. **b**, Structure of LiRecT predicted by RoseTTAFold (7). Extra segments predicted by RoseTTAFold that are absent from the cryo-EM structure are colored magenta. **c**, Structure of λ-Redβ predicted by RoseTTAFold. The helical bundle at the lower left is the C-terminal domain. **d**, Alignment of the cryo-EM (cyan) and RoseTTAFold (green/magenta) structures of LiRecT. **e**, Alignment of the cores of the LiRecT cryo-EM (cyan) and λ-Redβ RoseTTAFold (green) structures. Notice that λ-Redβ is missing the βA–βB insertion of LiRecT and has an extended loop between β5 and α3 (in place of αD and αE).

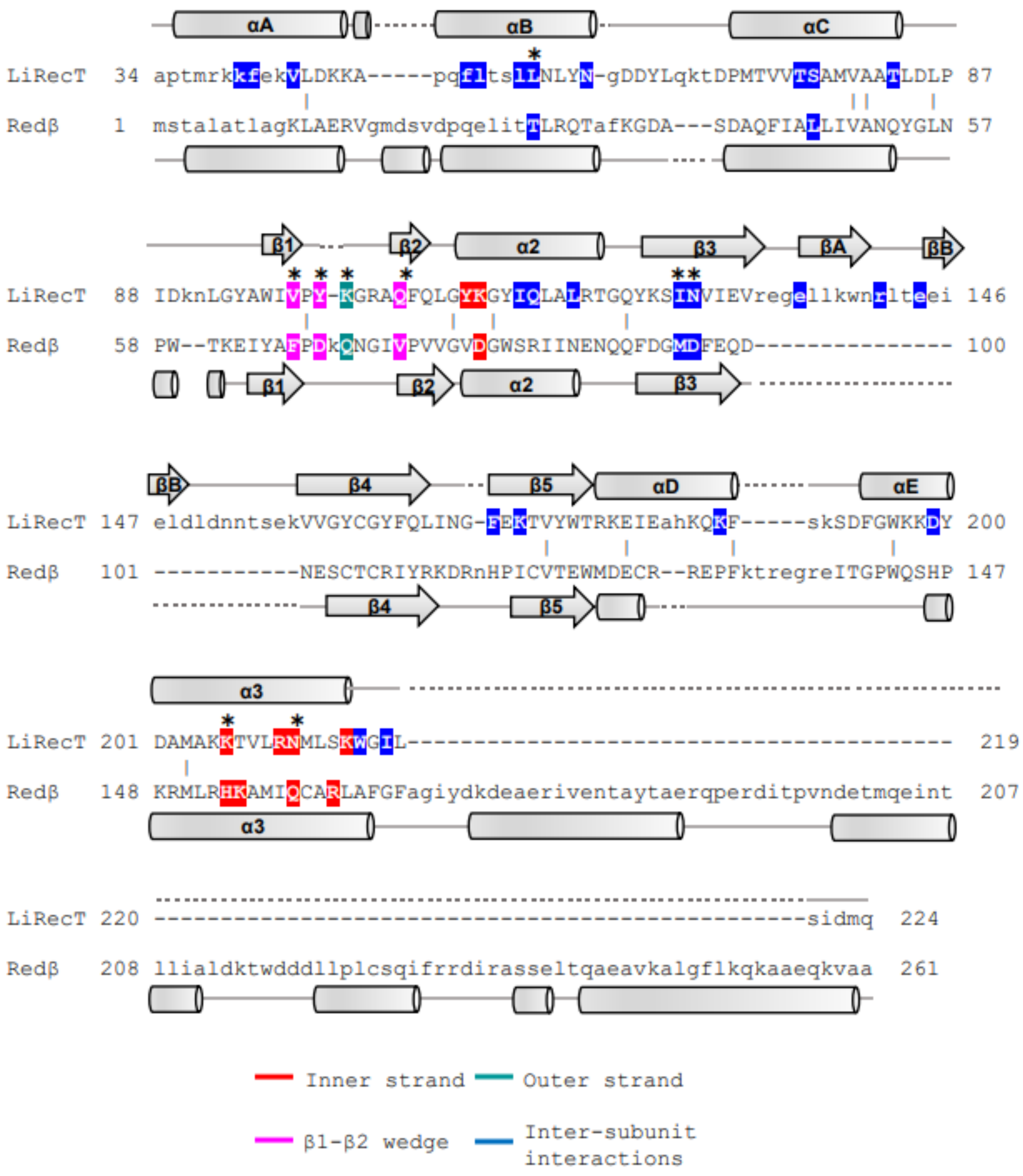

Supplementary Fig. 7 | Structure-based sequence alignment of LiRecT with a model of λ-Redβ predicted by RoseTTAFold. Structurally equivalent residues are in uppercase, structurally non-equivalent residues (including insertions) are in lowercase. Amino acid identities are indicated by vertical bars. The shading indicates amino acids of LiRecT that contact the inner strand (red), the outer strand (teal), the DNA from the β1-β2 hairpin insertion (violet), and form inter-subunit interactions (blue). The ∗ symbols indicate key functional residues of LiRecT and their predicted structurally conserved counterparts in λ-Redβ.

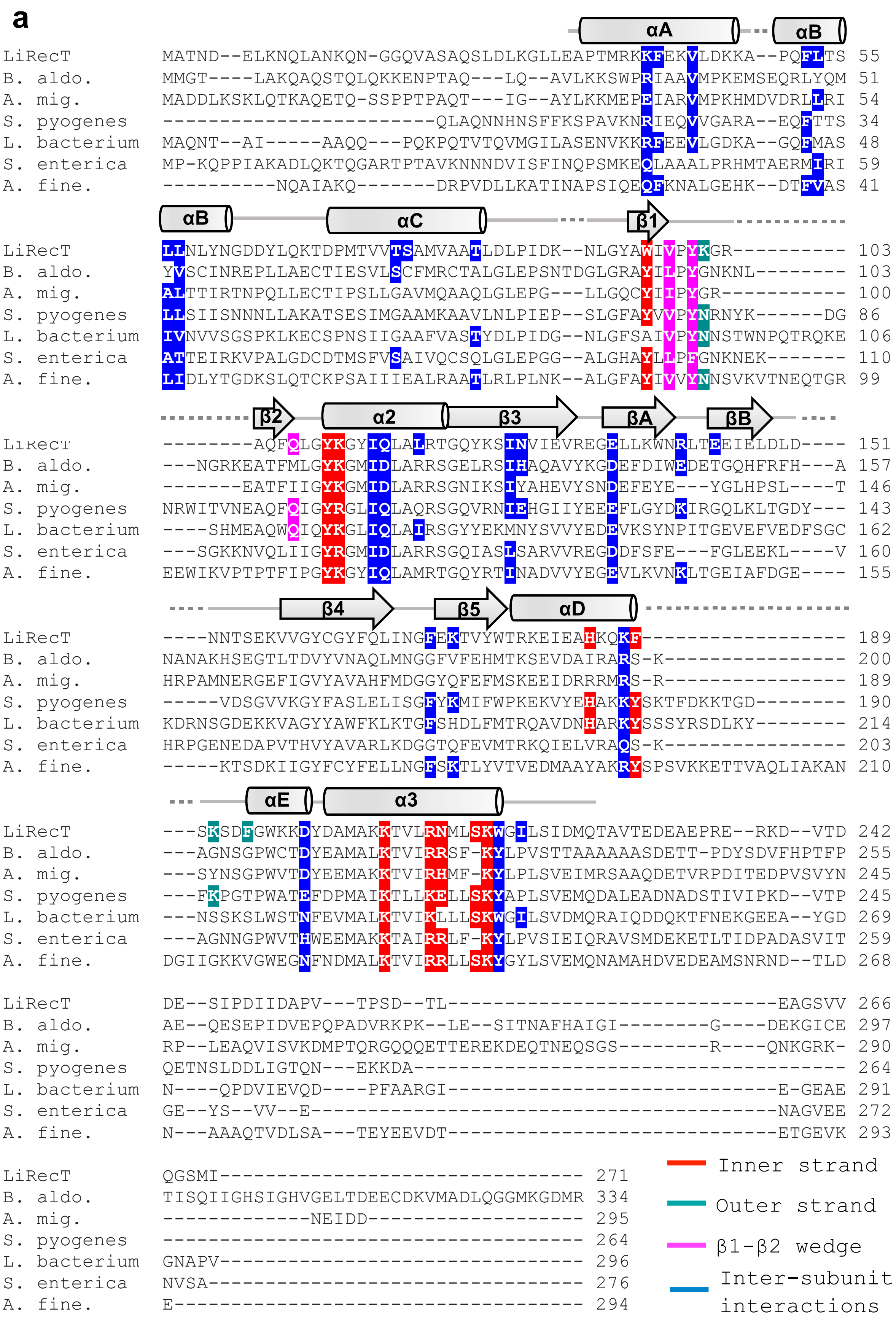
a
αA αB
LiRecT MATND--ELKNQLANKQN-GGQVASAQSLDLKGLLEAPTMRKKFEKVLDKKA--PQFLTS 55
B. aldo. MMGT----LAKQAQSTQLQKKENPTAQ---LQ--AVLKKSWPRIAAVMPKEMSEQRLYQM 51
A. mig. MADDLKSKLQTKAQETQ-SSPPTPAQT---IG--AYLKKMEPEIARVMPKHMDVDRLLRI 54
S. pyogenes ------------------------QLAQNNHNSFFKSPAVKNRIEQVVGARA--EQFTTS 34
L. bacterium MAQNT--AI-----AAQQ---PQKPQTVTQVMGILASENVKKRFEEVLGDKA--GQFMAS 48
S. enterica MP-KQPPIAKADLQKTQGARTPTAVKNNNDVISFINQPSMKEQLAAALPRHMTAERMIRI 59
A. fine. ----------NQAIAKQ-------DRPVDLLKATINAPSIQEQFKNALGEHK--DTFVAS 41
αB αC β1
LiRecT LLNLYNGDDYLQKTDPMTVVTSAMVAATLDLPIDK--NLGYAWIVPYKGR---------- 103
B. aldo. YVSCINREPLLAECTIESVLSCFMRCTALGLEPSNTDGLGRAYILPYGNKNL-------- 103
A. mig. ALTTIRTNPQLLECTIPSLLGAVMQAAQLGLEPG---LLGQCYIIPYGR----------- 100
S. pyogenes LLSIISNNNLLAKATSESIMGAAMKAAVLNLPIEP--SLGFAYVVPYNRNYK------DG 86
L. bacterium IVNVVSGSPKLKECSPNSIIGAAFVASTYDLPIDG--NLGFSAIVPYNNSTWNPQTRQKE 106
S. enterica ATTEIRKVPALGDCDTMSFVSAIVQCSQLGLEPGG--ALGHAYLLPFGNKNEK------- 110
A. fine. LIDLYTGDKSLQTCKPSAIIIEALRAATLRLPLNK--ALGFAYIVVYNNSVKVTNEQTGR 99
β2 α2 β3 βA βB
LiRecT --------AQFQLGYKGYIQLALRTGQYKSINVIEVREGELLKWNRLTEEIELDLD---- 151
B. aldo. ---NGRKEATFMLGYKGMIDLARRSGELRSIHAQAVYKGDEFDIWEDETGQHFRFH---A 157
A. mig. -------EATFIIGYKGMIDLARRSGNIKSIYAHEVYSNDEFEYE---YGLHPSL----T 146
S. pyogenes NRWITVNEAQFQIGYRGLIQLAQRSGQVRNIEHGIIYEEEFLGYDKIRGQLKLTGDY--- 143
L. bacterium ----SHMEAQWQIQYKGLIQLAIRSGYYEKMNYSVVYEDEVKSYNPITGEVEFVEDFSGC 162
S. enterica ---SGKKNVQLIIGYRGMIDLARRSGQIASLSARVVREGDDFSFE---FGLEEKL----V 160
A. fine. EEWIKVPTPTFIPGYKGYIQLAMRTGQYRTINADVVYEGEVLKVNKLTGEIAFDGE---- 155
β4 β5 αD
LiRecT ----NNTSEKVVGYCGYFQLINGFEKTVYWTRKEIEAHKQKF------------------ 189
B. aldo. NANAKHSEGTLTDVYVNAQLMNGGFVFEHMTKSEVDAIRARS-K---------------- 200
A. mig. HRPAMNERGEFIGVYAVAHFMDGGYQFEFMSKEEIDRRRMRS-R---------------- 189
S. pyogenes -----VDSGVVKGYFASLELISGFYKMIFWPKEKVYEHAKKYSKTFDKKTGD-------- 190
L. bacterium KDRNSGDEKKVAGYYAWFKLKTGFSHDLFMTRQAVDNHARKYSSSYRSDLKY-------- 214
S. enterica HRPGENEDAPVTHVYAVARLKDGGTQFEVMTRKQIELVRAQS-K---------------- 203
A. fine. -----KTSDKIIGYFCYFELLNGFSKTLYVTVEDMAAYAKRYSPSVKKETTVAQLIAKAN 210
αE α3
LiRecT ---SKSDFGWKKDYDAMAKKTVLRNMLSKWGILSIDMQTAVTEDEAEPRE--RKD--VTD 242
B. aldo. ---AGNSGPWCTDYEAMALKTVIRRSF-KYLPVSTTAAAAASDETT-PDYSDVFHPTFP 255
A. mig. ---SYNSGPWVTDYEEMAKKTVIRHMF-KYLPLSVEIMRSAAQDETVRPDITEDPVSVYN 245
S. pyogenes ---FKPGTPWATEFDPMAIKTLLKELLSKYAPLSVEMQDALEADNADSTIVIPKD--VTP 245
L. bacterium ---NSSKSLWSTNFEVMALKTVIKLLLSKWGILSVDMQRAIQDDQKTFNEKGEEA--YGD 269
S. enterica ---AGNNGPWVTHWEEMAKKTAIRRLF-KYLPVSIEIQRAVSMDEKETLTIDPADASVIT 259
A. fine. DGIIGKKVGWEGNFNDMALKTVIRRLLSKYGYLSVEMQNAMAHDVEDEAMSNRND--TLD 268
LiRecT DE--SIPDIIDAPV---TPSD--TL-----------------------------EAGSVV 266
B. aldo. AE--QESEPIDVEPQPADVRKPK--LE--SITNAFHAIGI--------G----DEKGICE 297
A. mig. RP--LEAQVISVKDMPTQRGQQQETTEREKDEQTNEQSGS--------R----QNKGRK- 290
S. pyogenes QETNSLDDLIGTQN---EKKDA--------------------------------------- 264
L. bacterium N----QPDVIEVQD----PFAARGI----------------------------E-GEAE 291
S. enterica GE--YS--VV--E---NAGVEE 272
A. fine. N---AAAQTVDLSA---TEYEEVDT-----------------------------ETGEVK 293
LiRecT QGSMI-------------------------------- 271
B. aldo. TISQIIGHSIGHVGELTDEECDKVMADLQGGMKGDMR 334
A. mig. -------------NEIDD------------------- 295
S. pyogenes ------------------------------------- 264
L. bacterium GNAPV-------------------------------- 296
S. enterica NVSA--------------------------------- 276
A. fine. E------------------------------------ 294
Inner strand
Outer strand
β1-β2 wedge
Inter-subunit interactions

b

	Li	Ba	Am	Sp	Lb	Se	Af
**LiRecT**	100	25.38	24.60	36.02	37.69	26.48	41.83
**B. aldo.**	25.38	100	44.06	23.75	22.26	25.38	24.02
**A. mig.**	24.60	44.06	100	27.51	25.40	24.60	23.05
**S. pyogenes**	36.02	23.75	27.51	100	34.90	36.02	31.54
**L. bacterium**	37.69	22.26	25.40	34.90	100	37.69	34.19
**S. Enterica**	26.48	36.60	43.75	24.57	23.64	100	23.58
**A. fine.**	41.83	24.02	23.05	31.54	34.19	41.83	100

Supplementary Fig. 8 | Position specific iterative blast alignment of seven LiRecT homologs. a, Multiple sequence alignment from Clustal-Omega (8). The homologs were chosen on the basis of having low to moderate levels of sequence identity with LiRecT and with one another (see panel b). Secondary structure elements of LiRecT are shown above the alignment. The shading indicates amino acids in LiRecT that contact the inner strand (red), the outer strand (teal), form the β1-β2 wedge (violet), and form inter-subunit interactions (blue). These residues are also shaded in the sequence of the homologs that contain a similar amino acid type at that position. **b,** Matrix showing the pair-wise sequence identities for all seven homologs. All homologs share from 23-44% pairwise sequence identity with LiRecT and with one another. The abbreviations to the left of each sequence correspond to: B. Aldo., *Bifidobacterium adolescentis* (WP_147538225.1); A. mig., *Aneurinibacillus migulanus* (WP_043070608.1); S. pyogenes, *Streptococcus pyogenes* (WP_115222948.1); L. bacterium, *Lachnospiraceae bacterium* (MBQ9437646.1); S. enterica, *Salmonella enterica* (EAO8776355.1); A. fine., *Alistipes finegoldii* (WP_014774661.1)

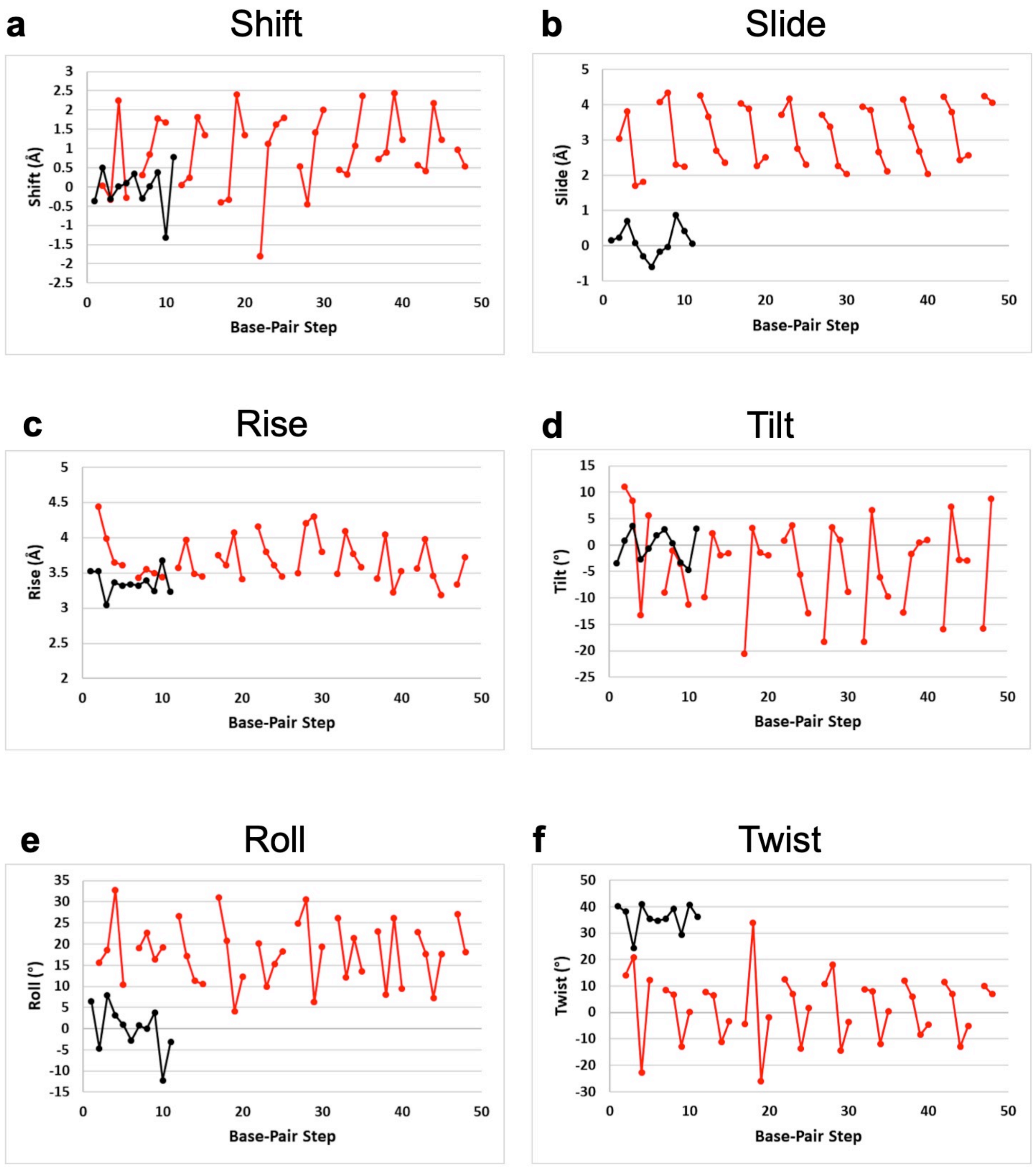

Supplementary Fig. 9 | Local base-pair step parameters of the annealed duplex bound to LiRecT. The parameters for the DNA bound to LiRecT (red lines) and B-form DNA from PDB code 1BNA (black lines) were calculated using the 3DNA program (9). Gaps in the red lines for LiRecT occur at every 5th base-pair step where consecutive base pairs are separated by ~9Å. The base-pair step number (n) on the x-axis indicates the base-pair step between the nth base and the (n+1)th base. Graphical representations of the parameters plotted in each graph (**a-f**) are provided at http://web.x3dna.org/public/images/bppar.jpg (9).

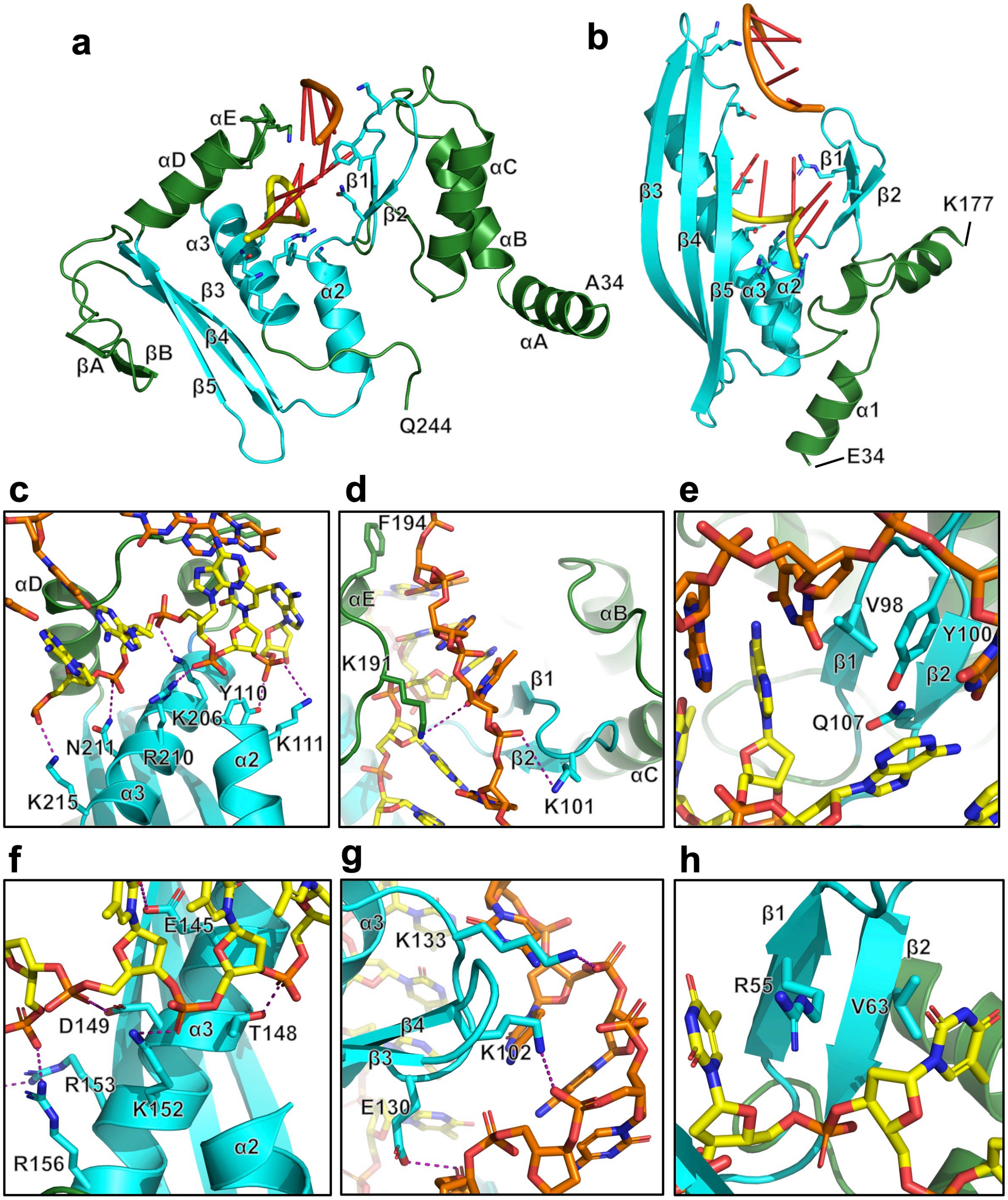

Supplementary Fig. 10 | Structural homology and DNA binding residues of LiRecT and RAD52. a, A monomer of LiRecT from the cryo-EM structure, with the core common to RAD52 colored in cyan. **b**, Structure of RAD52 with DNA bound to the inner site (PDB ID 5RXZ), with the ssDNA from a separate structure with ssDNA bound to the outer site (PDB ID 5XS0) superimposed (10). **c**, Residues in α2 and α3 of LiRecT contact the sugar phosphate backbone of the inner strand (yellow). **d**, Residues in the two loop regions of LiRecT at the outer portion of the groove contact the outer strand (orange). **e**, Residues from the β1-β2 hairpin that inserts into the bp at every 5th base pair step. **f-h,** The three corresponding regions of interaction are shown for RAD52 (10).

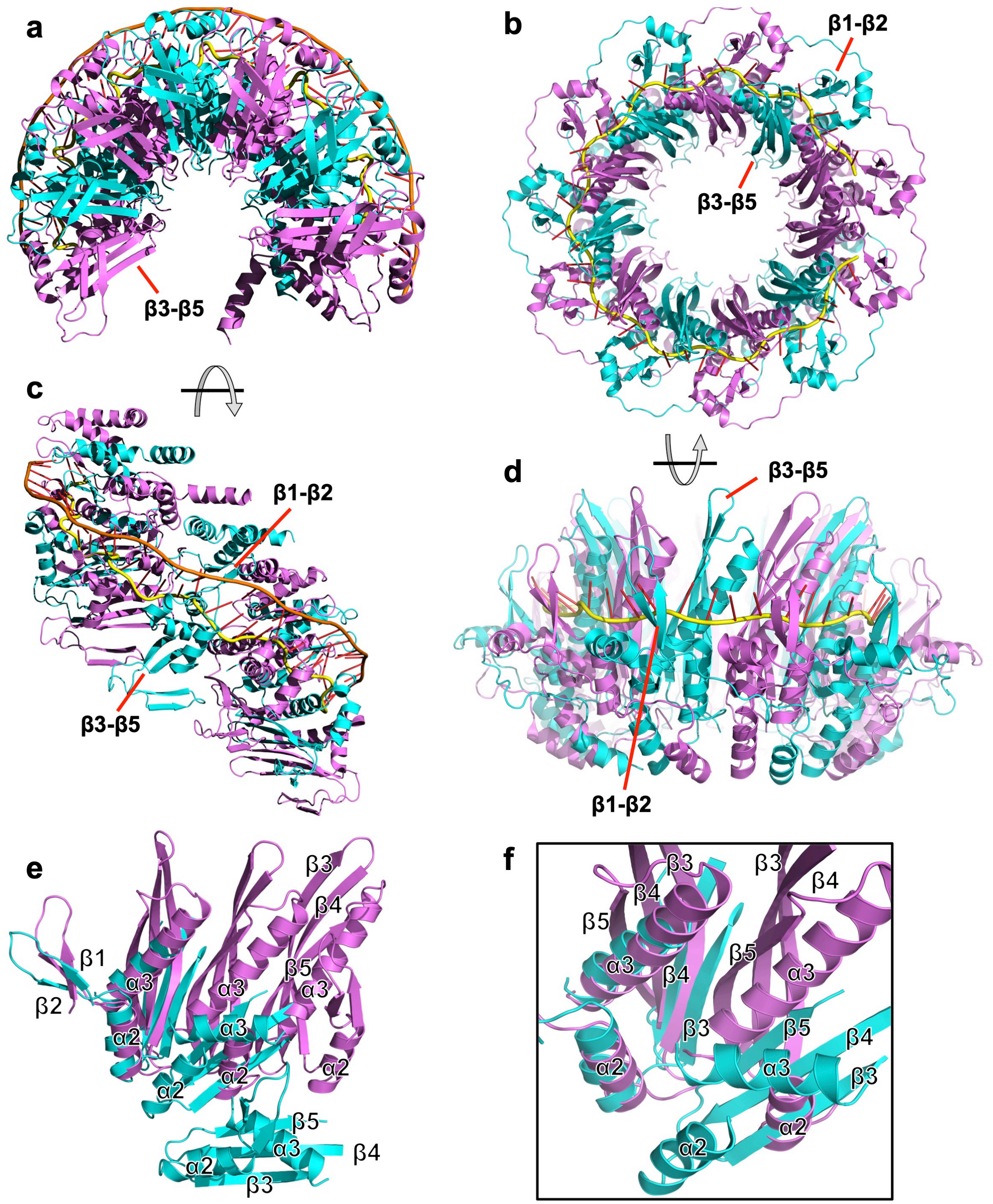

Supplementary Fig. 11 | Comparison of the inter-subunit packing in LiRecT and RAD52. a, View from the bottom of the LiRecT filament looking up the helical axis. Notice that the DNA is on the outer surface of the filament and the β3–β5 sheet is on the bottom surface (facing the reader). **b**, Top view of the closed 11-mer ring of RAD52 bound to a dT40 oligonucleotide (PDB ID 5XRZ; 10). Notice that the β3–β5 sheet is on the inner surface of the ring, and the β1–β2 hairpin is on the outer surface. **c, d,** Side views of panels a and b, respectively. Notice in panel c that the β1–β2 hairpin of LiRecT is above the DNA-binding groove, and the β3–β5 sheet is below it. Notice in panel d that the β1–β2 hairpin is on the outside of the DNA binding groove while the β3–β5 sheet is on the inside. **e**, The core portions of three subunits of RAD52 (violet) and LiRecT (cyan) are aligned by their left-most subunits, to show the reorientation in subunit packing to form the ring (violet) or the filament (cyan). **f**, Close-up view of the subunit interface shows that the two proteins pack into their oligomers using the same structural elements, β3 and α2 from the left subunit and β5 and α3 from the right subunit, but in slightly different orientations.

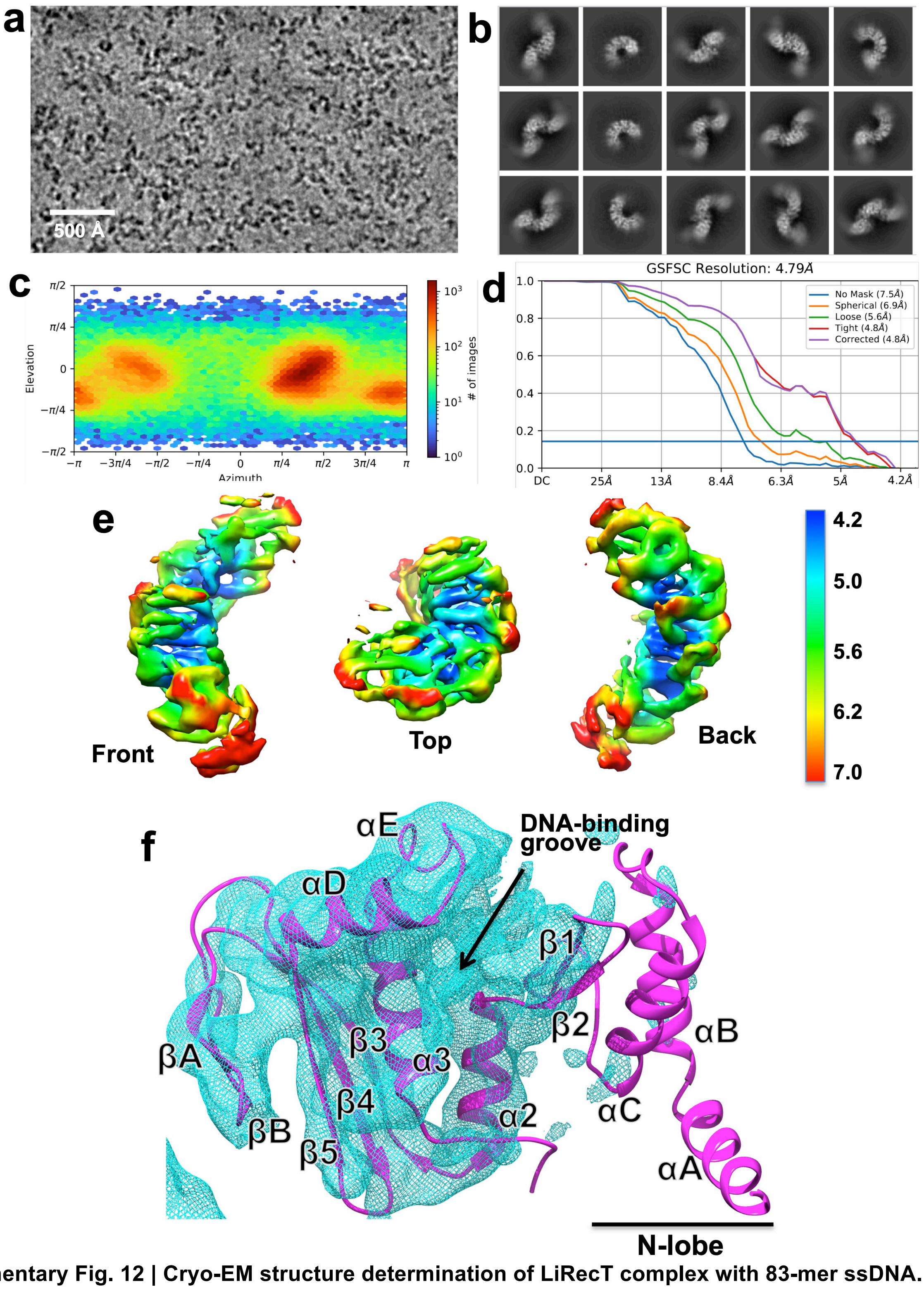

Supplementary Fig. 12 | Cryo-EM structure determination of LiRecT complex with 83-mer ssDNA. a, Krios K3 image at 81,000x. The image is one of 1619 that gave similar results. **b**, example 2D class averages. **c**, angular distribution of particles used for final reconstruction. **d**, resolution estimate by gold-standard Fourier Shell Correlation (FSC). **e**, final 3D reconstruction colored by local resolution estimate. **f**, CryoEM density drawn around a central subunit of the filament (Chain F). Notice that the density for the N-terminal helical bundle (N-lobe) is absent, and this region was not included in the final model. There was however strong density in the DNA binding groove that could not be interpreted. Figures were generated by cryoSPARC (2) and UCSF Chimera (3).

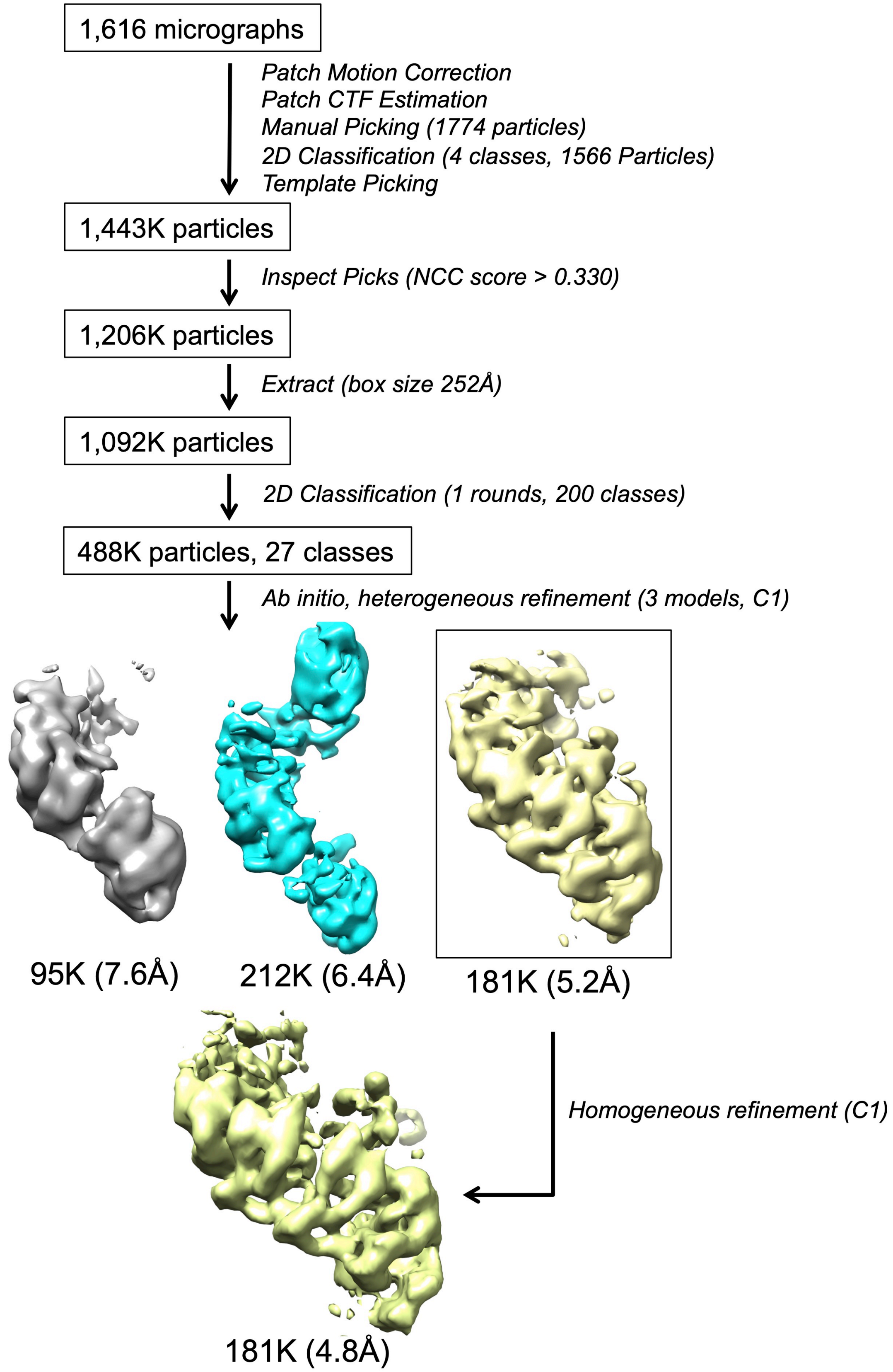

Supplementary Fig. 13 | Cryo-EM single particle workflow for complex of LiRecT with 83-mer ssDNA. The analysis was performed in cryoSPARC (2) and the figures were drawn with UCSF Chimera (3).

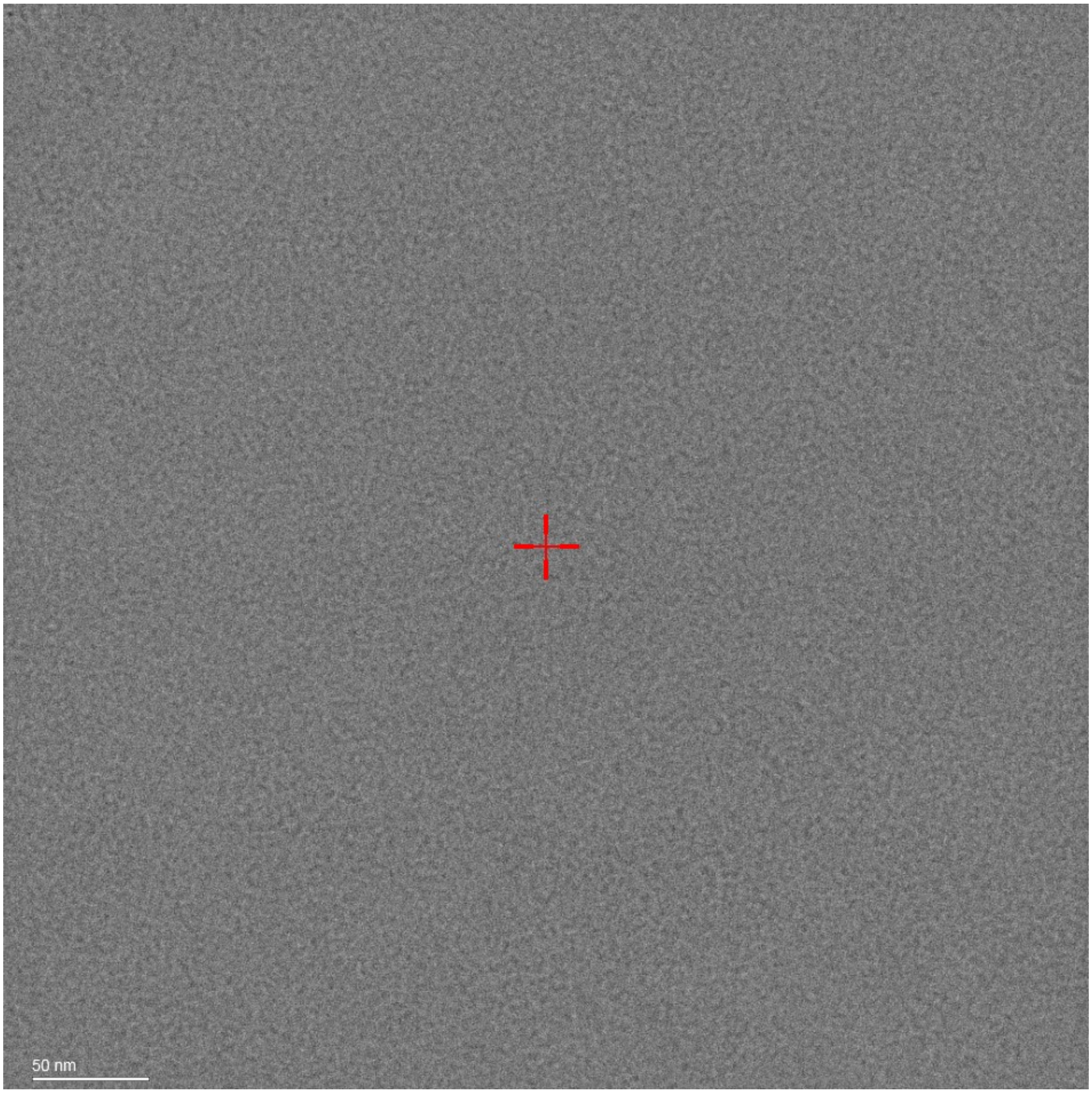

Supplementary Fig. 14 | Cryo-EM image of LiRecT protein without DNA. 1.4 mg/ml LiRecT in cryo-EM buffer was imaged at 93,000x (pixel size 1.13Å) at a defocus of -3 μm using a 200 kev Glacios instrument and a Falcon 3 direct electron detector. Notice that no visible filaments are formed, in contrast to the particles formed when 83mer ssDNA is added. The image shown is one of eight that all gave similar results.

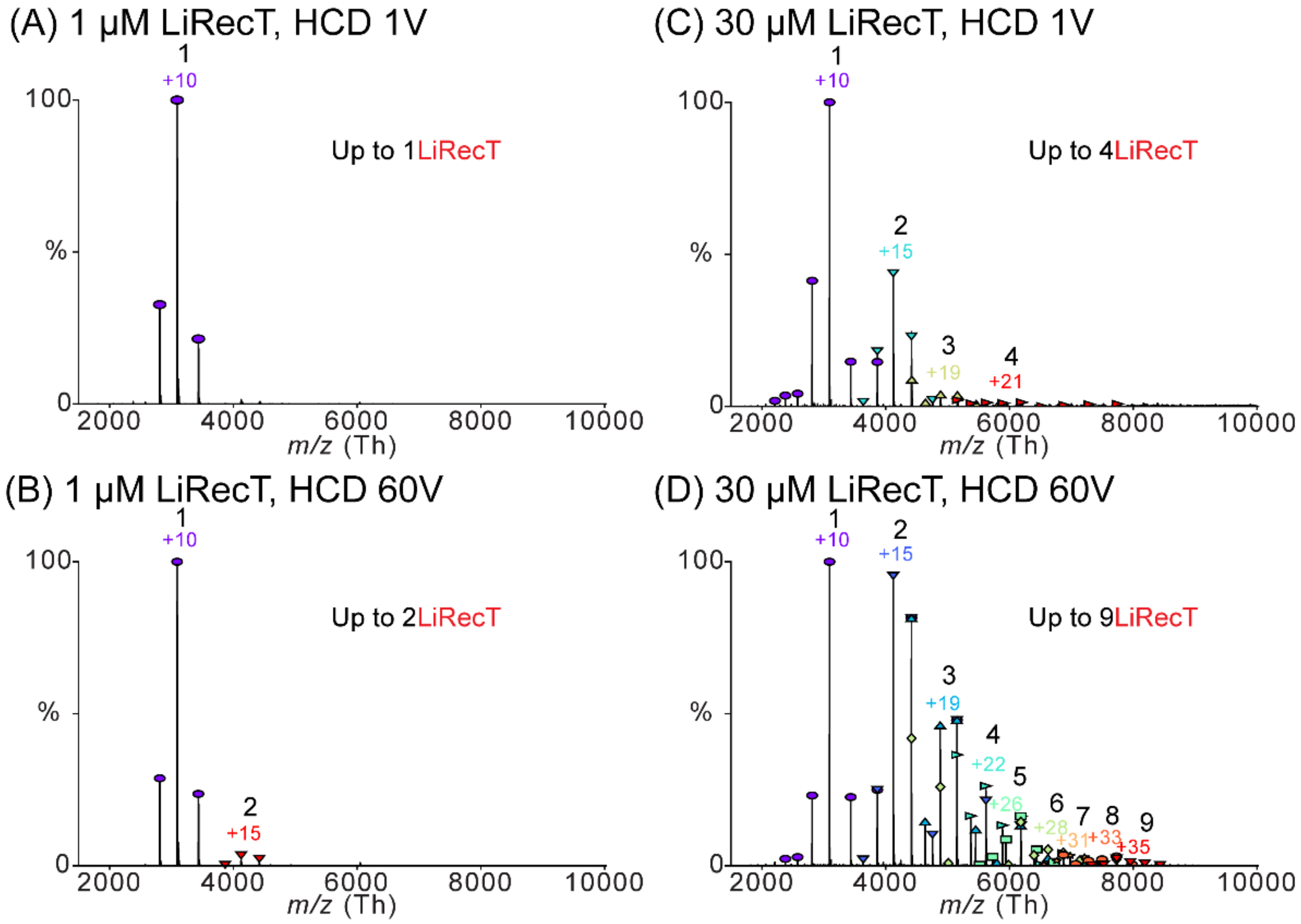

Supplementary Fig. 15 | Native mass spectra of LiRecT protein alone. Protein was dialyzed into 100 mM ammonium acetate and injected at 1 μM (A & B) or 30 μM (C & D). The effect of HCD is compared for 1 μM LiRecT with (A) 1 V HCD versus (B) 60 V HCD, and for 30 μM LiRecT with (C) 1 V HCD versus (D) 60 V HCD. Within each spectrum the charge state distribution for each species is indicated by the numbers in different colors. The number in black above each charge state indicates the LiRecT oligomeric state for each distribution. Each species (monomer through 9-mer) is identified with a different colored symbol, as defined by the numbers above each charge state.

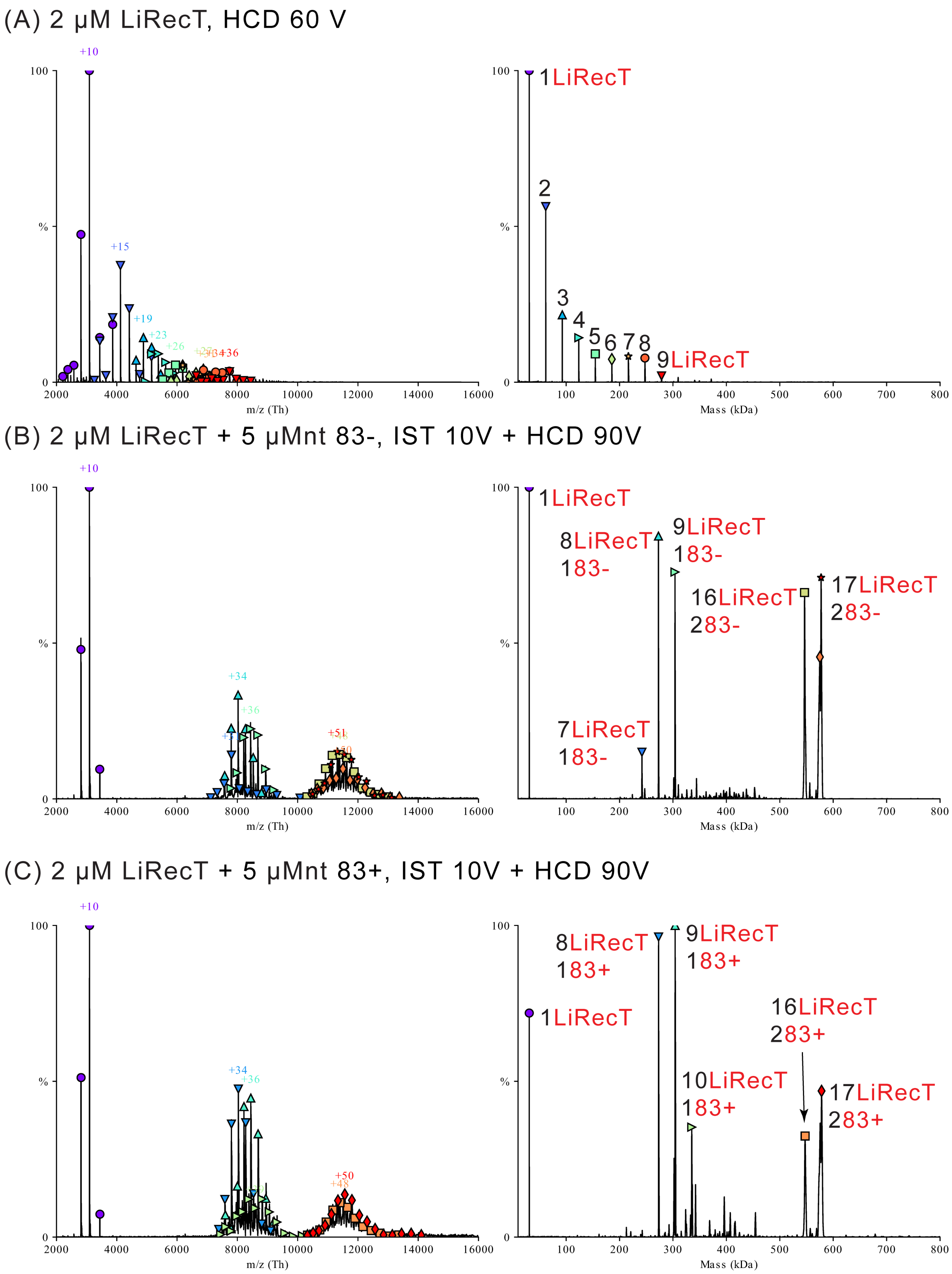

(A) 2 µM LiRecT, HCD 60 V
+10
+15
+19
+23
+26
+34
+36
m/z (Th)
Mass (kDa)
1LiRecT
2
3
4
5
6
7
8
9LiRecT
(B) 2 µM LiRecT + 5 µMnt 83-, IST 10V + HCD 90V
+10
+34
+36
+51
m/z (Th)
Mass (kDa)
1LiRecT
8LiRecT
183-
9LiRecT
183-
16LiRecT
283-
17LiRecT
283-
7LiRecT
183-
(C) 2 µM LiRecT + 5 µMnt 83+, IST 10V + HCD 90V
+10
+34
+36
+50
+48
m/z (Th)
Mass (kDa)
8LiRecT
183+
9LiRecT
183+
1LiRecT
16LiRecT
283+
10LiRecT
183+
17LiRecT
283+

(D) 2 µM LiRecT + 5 µMnt 80-, IST 10V + HCD 90V

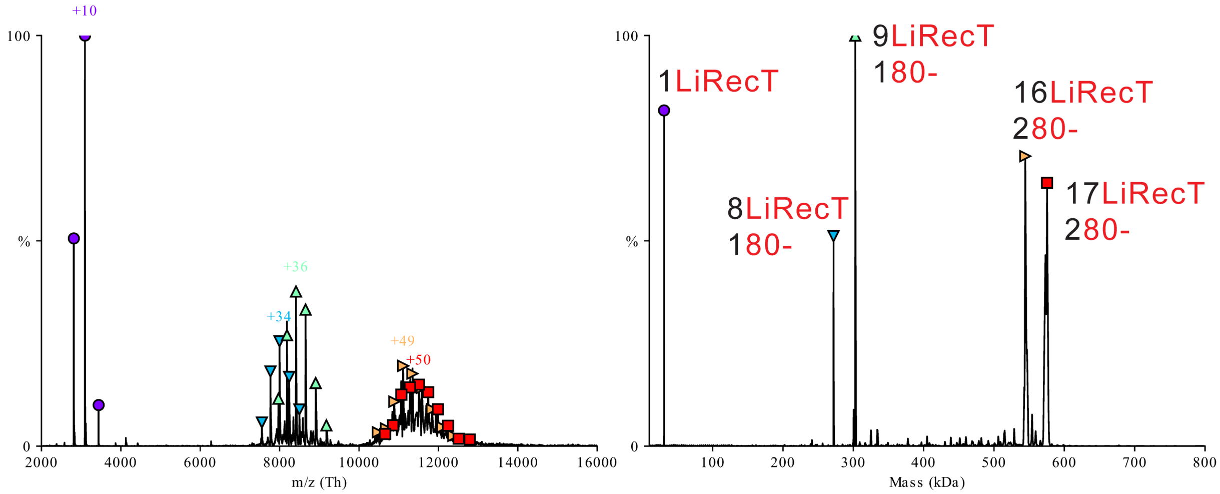

(E) 2 µM LiRecT + 5 µMnt 80+, IST 10V + HCD 90V

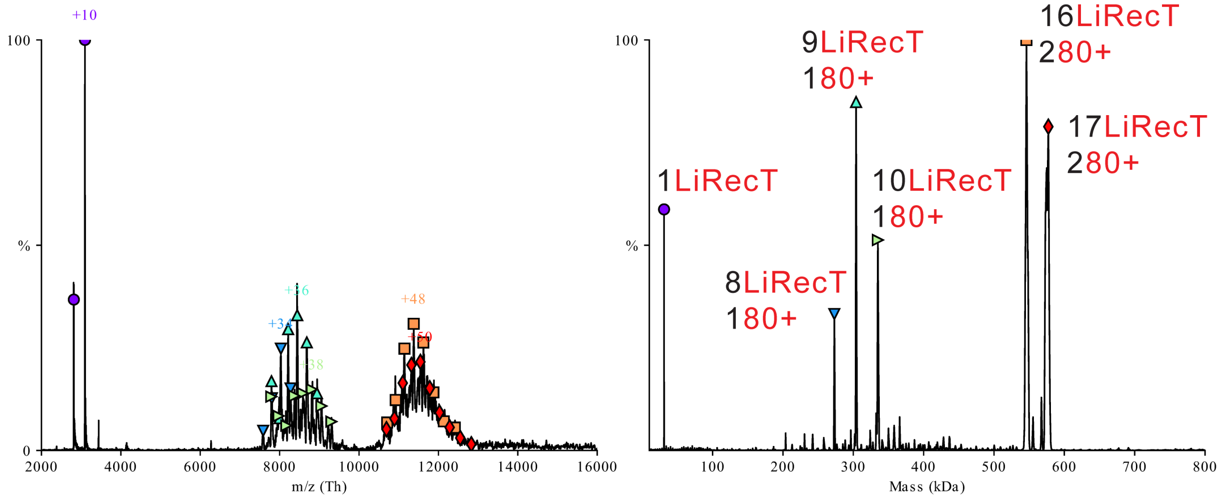

(F) 2 µM LiRecT + 5 µMnt 75-, IST 10V + HCD 90V

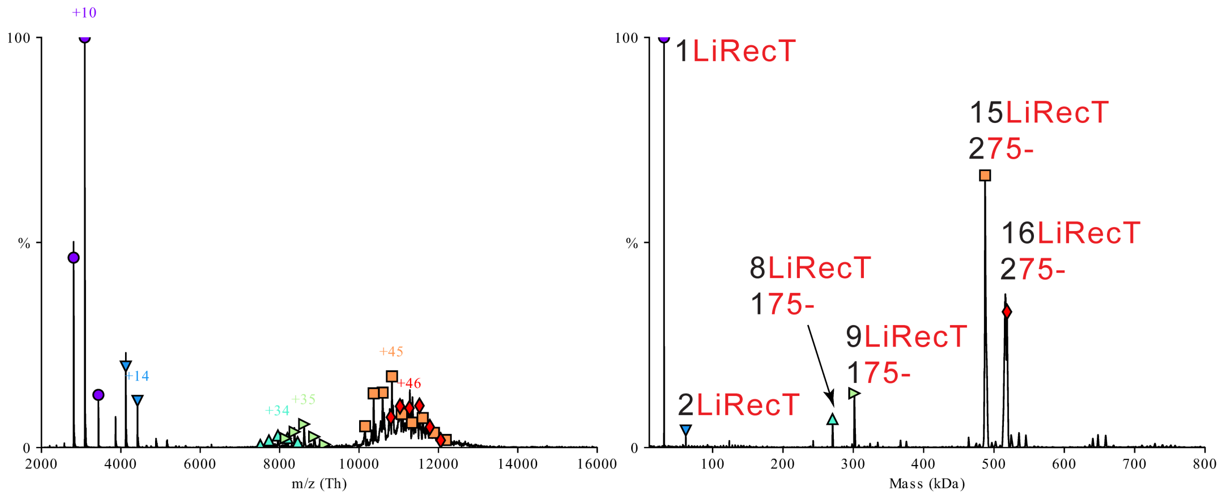

(G) 2 μM LiRecT + 5 μMnt 75+, IST 10V + HCD 90V

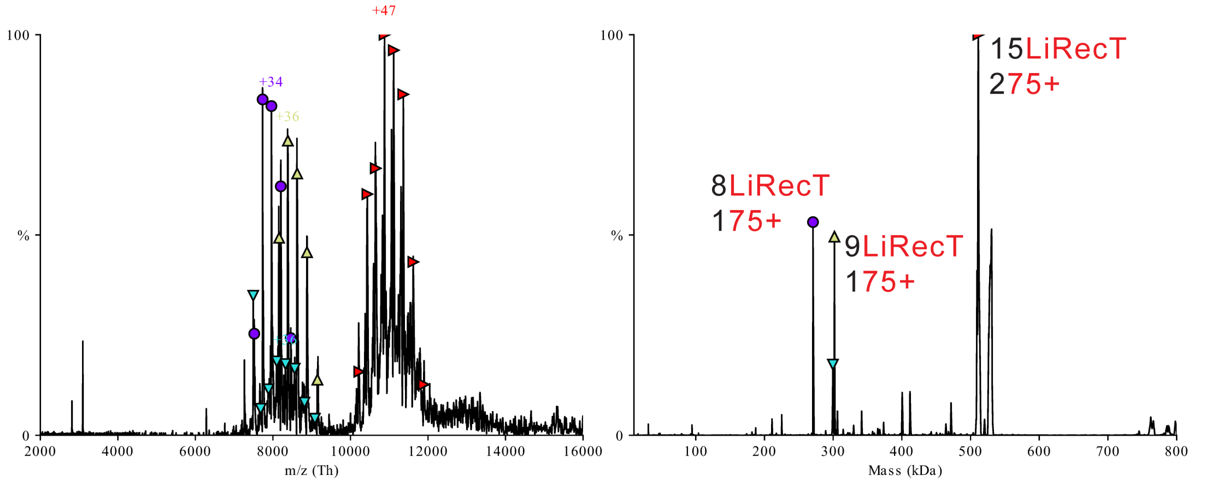

(H) 2 μM LiRecT + 5 μMnt 83-:83+, IST 10V + HCD 90V

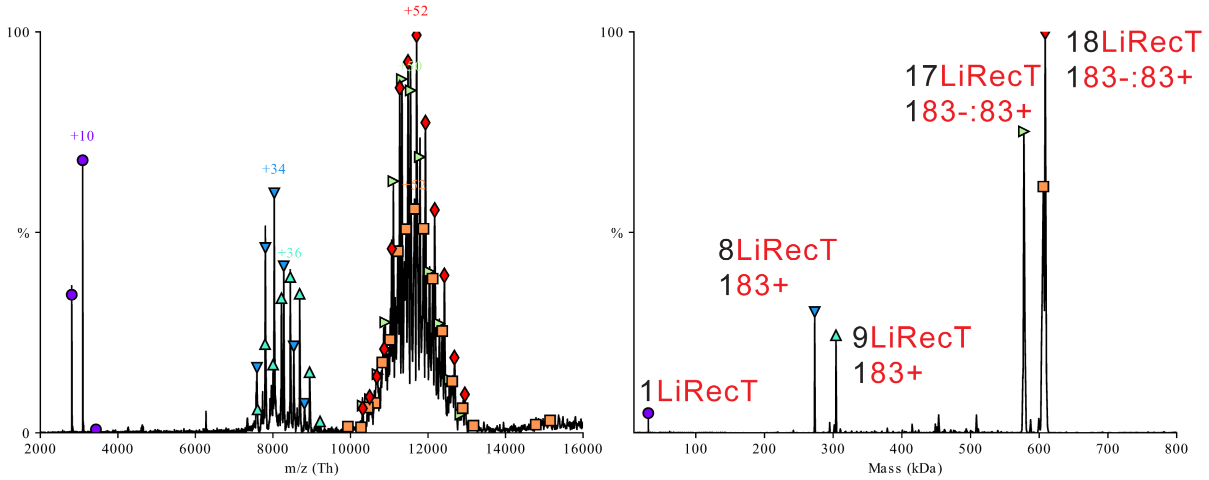

(I) 2 μM LiRecT + 5 μMnt 80-:80+, IST 10V + HCD 90V

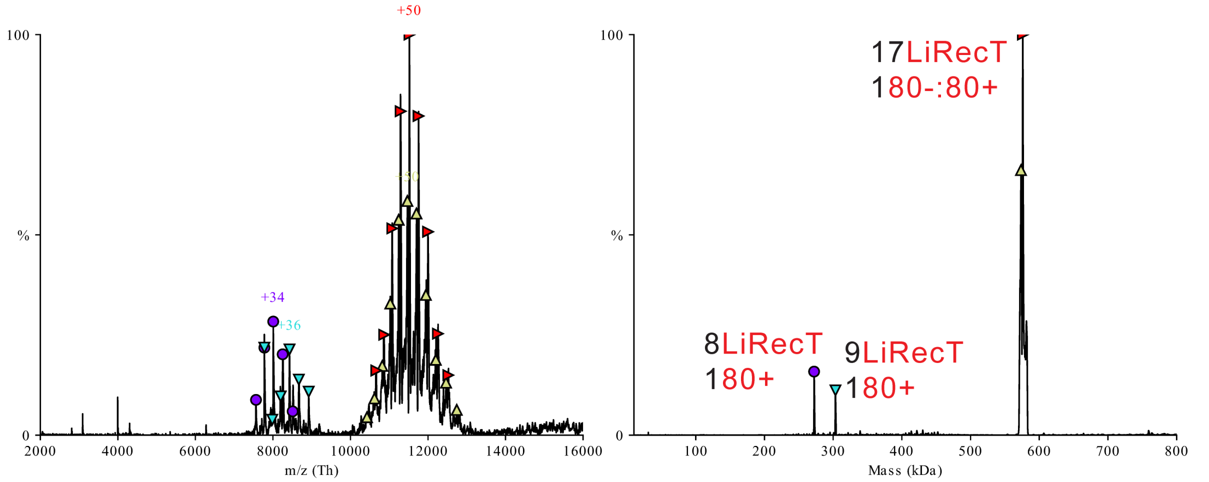

(J) 2 μM LiRecT + 5 μMnt 75-:75+, IST 10V + HCD 90V

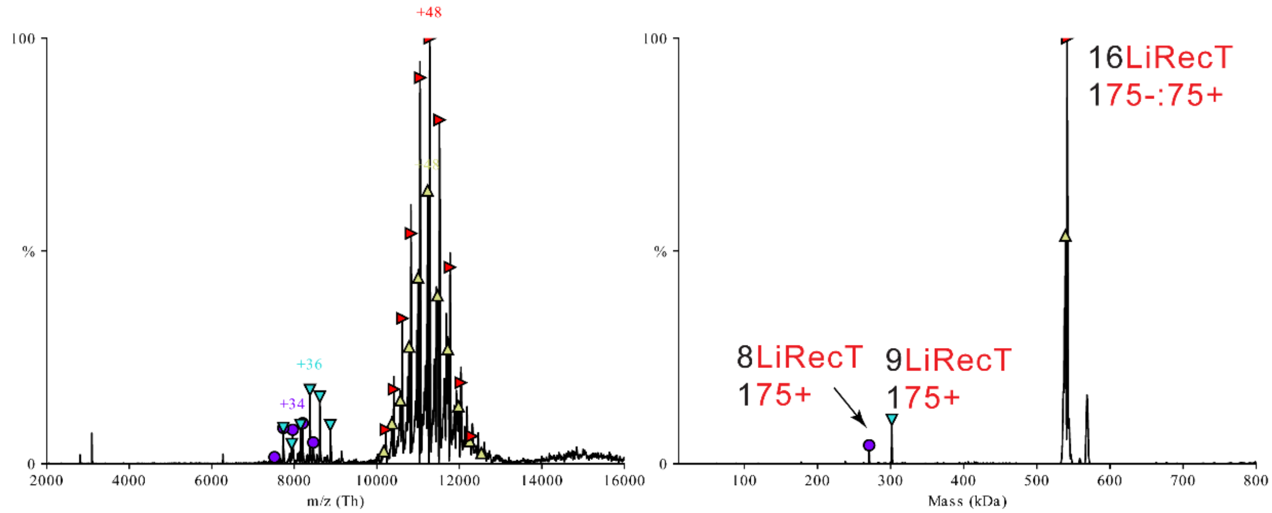

Supplementary Fig. 16 | Mass spectra (left) and zero-charge mass spectra (right) of 2 μM LiRecT mixed with different lengths and combinations of DNA . (A) LiRecT protein alone, (B) 83-, (C) 83+, (D) 80-, (E) 80+, (F) 75-, (G) 75+, (H) 83-:83+, (I) 80-:80+, (J) 75-:75+. For panels H, I, and J, two oligos were added to the protein sequentially (first:last), as described in Materials and Methods. Integrated values of the relative amounts of each species in these spectra were used to generate the data plotted in Fig. 6. All DNAs were added at a concentration of 5 μM nucleotides. The colored circles and triangles indicate the associated peak(s) for each different species, as defined in right panels with name of the species (protein or DNA) in red font and number of copies of each species in black font.

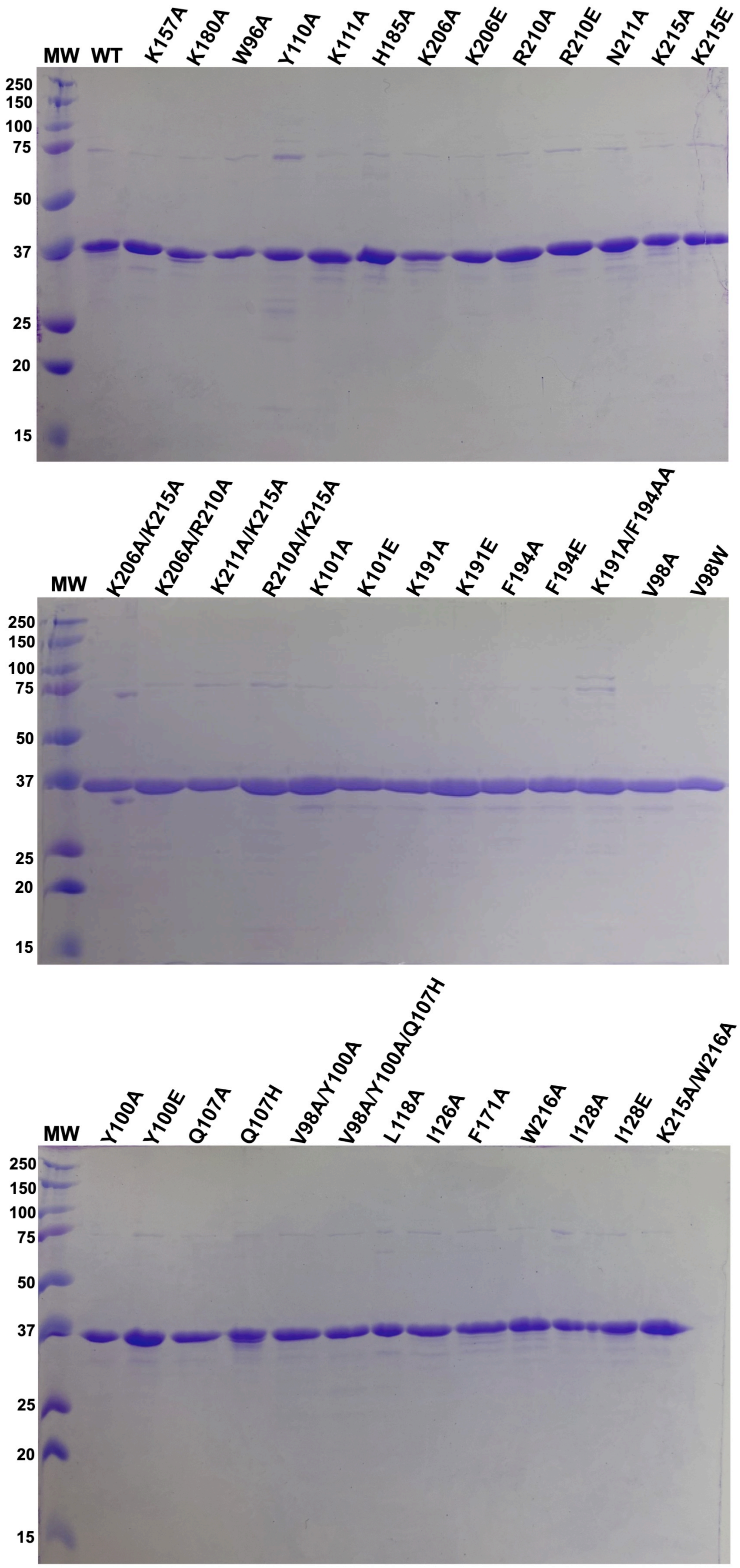

Supplementary Fig. 17 | SDS-PAGE of LiRecT protein variants used for the mutational analysis. Each lane contains 1.4 µg of purified protein. The 12.5% gel was stained with Coomassie brilliant blue R250. The variants appear in the same order as they are presented in Fig. 8 (by location in the structure). Each purified protein was analyzed at least twice with very similar results. Source data are provided as a Source Data File.

Supplementary Table 1. Cryo-EM Data Collection and Refinement Statistics[1]

	LiRecT + 83-:83+ (Annealed Duplex)	LiRecT + 83- (ssDNA)
EMDB Code	EMD-26434	EMD-26437
PDB Code	7UB2	7UBB
Magnification	81,000x	81,000x
Voltage (Kv)	300	300
Dose (e-/Å^2)	66	65
Defocus range (μm)	-3.5 to -1.0	-3.5 to -1.0
Pixel size (data collection) (Å)	0.899	0.4595
Pixel size (reconstruction) (Å)	0.899	0.899
No. of movies collected	2038	1619
Symmetry imposed	C1	C1
Particle Images	270,721	180,965
Resolution (0.143 FSC, tight mask) (Å)	3.41	4.79
r.m.s. deviation bond lengths (Å)	0.002	---
r.m.s. deviation bond angles (Å)	0.515	---
MolProbability Score	1.23	---
Clashscore	4.49	---
C-beta outliers (%)	0.00	---
Rotamer outliers (%)	0.60	---
CaBLAM outliers (%)	0.53	---
Ramachandran favored (%)	98.94	---
Ramachandran allowed (%)	1.06	---
Ramachandran outliers (%)	0.00	---

[1]The structure with ssDNA was limited to rigid body refinement

Supplementary Table 2. Data from native MS used for the heat map plot of LiRecT-DNA species in Fig. 6 and derived from spectra in Supplementary Figs. 15 & 16. Each table compares, for the indicated line in Fig. 6, the experimental mass of each peak in the deconvolved spectrum with the mass determined for each assigned complex.

Fig. 6, line 1 (UHMR1_nESI_LiRecT_2uM_HCD60)

Relative Intensity	Experimental Mass (Da)	Determined Mass (Da)	Difference (Da)	Species
100	30902	30901.79	0	1[LiRecT]
56	61808	61803.58	4	2[LiRecT]
22	92776	92705.37	71	3[LiRecT]
14	123685	123607.16	78	4[LiRecT]
9	154612	154508.96	103	5[LiRecT]
7	185559	185410.75	148	6[LiRecT]
8	216518	216312.54	205	7[LiRecT]
8	247479	247214.33	265	8[LiRecT]
2	278633	278116.12	517	9[LiRecT]

Fig. 6, line 2 (UHMR1_nESI_LiRecT_2uM_83-_5uMnt_IST10_HCD90)

Relative Intensity	Experimental Mass (Da)	Determined Mass (Da)	Difference (Da)	Species
100	30900	30901.79	-2	1[LiRecT]
15	241920	241744.24	176	7[LiRecT]1[83-]
84	272810	272646.03	164	8[LiRecT]1[83-]
73	303770	303547.82	222	9[LiRecT]1[83-]
66	546250	545292.06	958	16[LiRecT]2[83-]
71	577300	576193.85	1106	17[LiRecT]2[83-]

Fig. 6, line 3 (UHMR1_nESI_LiRecT_2uM_83+_5uMnt_IST10_HCD90)

Relative Intensity	Experimental Mass (Da)	Determined Mass (Da)	Difference (Da)	Species
72	30960	30901.79	58	1[LiRecT]
96	273110	272934.13	176	8[LiRecT]1[83+]
100	304050	303835.92	214	9[LiRecT]1[83+]
35	335120	334737.71	382	10[LiRecT]1[83+]
32	547380	545868.26	1512	16[LiRecT]2[83+]
47	578180	576770.05	1410	17[LiRecT]2[83+]

Fig. 6, line 4 (UHMR1_nESI_LiRecT_2uM_80-_5uMnt_IST10_HCD90)

Relative Intensity	Experimental Mass (Da)	Determined Mass (Da)	Difference (Da)	Species
82	30960	30901.79	58	1[LiRecT]
51	271880	271708.43	172	8[LiRecT]1[80-]
100	302820	302610.22	210	9[LiRecT]1[80-]
71	544460	543416.86	1043	16[LiRecT]2[80-]
64	575420	574318.65	1101	17[LiRecT]2[80-]

Fig. 4, line 5 (UHMR1_nESI_LiRecT_2uM_80+_5uMnt_IST10_HCD90)

Relative Intensity	Experimental Mass (Da)	Determined Mass (Da)	Difference (Da)	Species
59	30960	30901.79	58	1[LiRecT]
33	273050	272018.53	1031	8[LiRecT]1[80+]
85	303940	302920.32	1020	9[LiRecT]1[80+]
51	334910	333822.11	1088	10[LiRecT]1[80+]
100	546080	544037.06	2043	16[LiRecT]2[80+]
79	577250	574938.85	2311	17[LiRecT]2[80+]

Fig. 6, line 6 (UHMR1_nESI_LiRecT_2uM_75-_5uMnt_IST10_HCD90)

Relative	Experimental	Determined	Difference	Species

Intensity	Mass (Da)	Mass (Da)	(Da)	
100	30900	30901.79	-2	1[LiRecT]
4	61860	61803.58	56	2[LiRecT]
7	270470	270184.43	286	8[LiRecT]1[75-]
13	301540	301086.22	454	9[LiRecT]1[75-]
66	487290	486496.97	793	15[LiRecT]1[75-]
33	518440	517398.76	1041	16[LiRecT]1[75-]

Fig. 6, line 7 (UHMR1_nESI_LiRecT_2uM_75+_5uMnt_IST10_HCD90)

Relative Intensity	Experimental Mass (Da)	Determined Mass (Da)	Difference (Da)	Species
53	270640	270454.53	185	8[LiRecT]1[75+]
18	299620	301356.32	-1736	9[LiRecT]1[75+]
50	301850	301356.32	494	9[LiRecT]1[75+]
100	511290	510007.27	1283	15[LiRecT]2[75+]

Fig. 6, line 8 (UHMR1_nESI_LiRecT_2uM_83-:83+_5uMnt_IST10_HCD90)

Relative Intensity	Experimental Mass (Da)	Determined Mass (Da)	Difference (Da)	Species
5	30900	30901.79	-2	1[LiRecT]
30	273150	272934.13	216	8[LiRecT]1[83+]
24	304090	303835.92	254	9[LiRecT]1[83+]
75	577550	576481.95	1068	17[LiRecT]1[83-]1[83+]
100	608620	607383.74	1236	18[LiRecT]1[83-]1[83+]

Fig. 6, line 9 (UHMR1_nESI_LiRecT_2uM_80-:80+_5uMnt_IST10_HCD90)

Relative Intensity	Experimental Mass (Da)	Determined Mass (Da)	Difference (Da)	Species
16	272240	272018.53	221	8[LiRecT]1[80+]
11	303350	302920.32	430	9[LiRecT]1[80+]
100	575970	574628.75	1341	17[LiRecT]1[80-]1[80+]

Fig. 6, line 10 (UHMR1_nESI_LiRecT_2uM_75-:75+_5uMnt_tip2_IST10_HCD90)

Relative Intensity	Experimental Mass (Da)	Determined Mass (Da)	Difference (Da)	Species
4	270660	270454.53	205	8[LiRecT]1[75+]
10	301600	301356.32	244	9[LiRecT]1[75+]
100	541420	540638.96	781	16[LiRecT]1[75-]1[75+]

Supplementary Table 3. Product information for materials used in this study

Item name	Vendor	Catalog Number
Acrylamide/bis-Acrylamide	Research Products International	A11410-1000.0
Amicon Ultra-4 centrifugal Filter Unit	Millipore Sigma	UFC8010
Ammonium acetate (crystalline ACS)	Fisher Scienfific	A637-500
Ammonium persulfate	Fisher Bioreagents by Fisher Scientific™	BP179-25
BL21-AI cells	Invitrogen/Thermo Scientific	C607003
Boric Acid	Fisher Chemical	A73-500
Calcium chloride dihydrate (ACS)	Fisher Chemical	C79-500
Coomassie Brilliant Blue R-250	Bio-Rad	1610406
dNTPs	Invitrogen/Thermo Fisher Scientific	10297018
DpnI	New England Biolabs	10113063
Ethylenediaminetetraacetic acid	Fisher Scientific	BP118-500
Glacial Acetic Acid	Fisher Chemical	A38-212
Glycerol	Thermo Fisher Scientific	G33-4
HisTrap Fast Flow 5 ml	Cytiva	17-5255-01
HiTrap Q Fast Flow 5 ml	Cytiva	17-5156-01
Hydrochloric Acid	Fisher Chemical	A144-500
Imidazole (ACS Certified)	Fisher Chemical	O3196-500
IPTG	Research Products International	156000-100.0
Kanamycin sulfate	Fisher Bioreagents by Fisher Scientific™	25389-94-0
Laemmli sample buffer (4X)	Bio-Rad	1610747
L-(+)-Arabinose	Research Products International	A51000-500.0
LB Broth, granulated, Lennox	Research Products International	L24066-1000.0
LB Agar, low salt, granulated Lennox	Research Products International	L24033-1000.0
Leupeptin (90%, synthetic)	Acros Organics/Thermo Scientific	328350050
Lysozyme	MP Biomedicals	100831
Magnesium chloride hexahydrate	Fisher Scientific	M33-500
Manganese chloride tetrahydrate	Fisher Chemical	M87-500
Methanol	Fisher Bioreagents by Fisher Scientific™	BP1105-4
Micro Bio-Spin P-6 gel columns	Bio-Rad	7326221
MOPS	Sigma	M1254-250G
n-dodecyl-β-D-maltopyranoside	Anatrace	D310
Ni-NTA Spin Columns	Qiagen	31014
Orange G for NA electrophoresis	Sigma Life Sciences	O3756-25G
PD10-Desalting columns	Cytiva	170851-01
Pfu Turbo	Agilent Technologies	600252-52
Pfu Turbo Cx Hotstart DNA	Agilent Technologies	600412-51
PMSF	Research Products International	P20270-1.0
Potassium acetate (crystalline/ACS)	Fisher Chemical	P171-500
Potassium chloride (white crystals)	Fisher Bioreagents by Fisher Scientific™	BP366-1
Potassium phosphate monobasic	Fisher Chemical	P285-500
QIAprep® Spin Miniprep Kit	Qiagen	27106
Quantifoil R1.2/1.3 AU 300 mesh	Electron Microscopy Sciences	Q250-AR2
Reagent alcohol	Fisher Chemical	A962-4
SeaKem® LE Agarose	Lonza	50004
Sodium chloride	Fisher Chemical	S271-3
Sodium dodecyl sulfate, white poweder	Fisher Bioreagents by Fisher Scientific™	BP166-500
Sodium hydroxide (10N)	Fisher Chemical	SS255-1
Sodium phosphate monobasic monohydrate	Fisher Chemical	S369-500
Sucrose (crystalline certified ACS)	Fisher Chemical	S5-3
TEMED (electrophoresis grade)	Fisher Bioreagents by Fisher Scientific™	BP150-20
Thrombin protease	Cytiva	27084601
Tris/Glycine/SDS Buffer (10X)	Bio-Rad	1610772
Tris base Ultra Pure	Research Products International	T60040-100.0
Standard Vitrobot filter paper	Ted Pella	47000-100
Vivaspin 20 concentrator MWCO 10 kDa	Millipore Sigma	Z614610

Supplementary Table 4. Sequences of oligonucleotides used in this study.[1]

	Oligonucleotide	Sequence
native MS-gel shift	5'-Cy5 50mer	CCATCCGCAAAAATCGAGCTATGCAGGGCGATTCTGCTCTAAGCCATCCG
	5'-Cy3 50mer	GCGGATGGCTTAGAGCAGAATCGCCCTGCATAGCTCGATTTTTGCGGATG
	83-	TTGCATATTTAAAACATGTTGAGCTACAGCACCAGATTCAGCAATTAAGCTCTAAGCCATCCGCAAAAATGACCTCTTATCAA
	83+	TTGATAAGAGGTCATTTTTGCGGATGGCTTAGAGCTTAATTGCTGAATCTGGTGCTGTAGCTCAACATGTTTTAAATATGCAA
	80-	CATATTTAAAACATGTTGAGCTACAGCACCAGATTCAGCAATTAAGCTCTAAGCCATCCGCAAAAATGACCTCTTATCAA
	80+	TTGATAAGAGGTCATTTTTGCGGATGGCTTAGAGCTTAATTGCTGAATCTGGTGCTGTAGCTCAACATGTTTTAAATATG
	75-	TTAAAACATGTTGAGCTACAGCACCAGATTCAGCAATTAAGCTCTAAGCCATCCGCAAAAATGACCTCTTATCAA
	75+	TTGATAAGAGGTCATTTTTGCGGATGGCTTAGAGCTTAATTGCTGAATCTGGTGCTGTAGCTCAACATGTTTTAA
Mutagenesis – sequencing, controls and inner strand	T7 promoter primer	TAATACGACTCACTATAGGG
	T7 terminator primer	GCTAGTTATTGCTCAGCGG
	K157A for	GACAACAATACAAGTGAA**GCA**GTTGTTGGCTACTGTGGC
	K157A rev	GCCACAGTAGCCAACAACTGCTTCACTTGTATTGTTGTC
	K180A for	CGGTCTATTGGACTCGT**GCA**GAAATAGAAGCGCATAAAC
	K180A rev	GTTTATGCGCTTCTATTTCTGCACGAGTCCAATAGACCG
	W96A for	CGACAAAAATTTAGGTTATGCG**GCG**ATTGTTCCTTACAAAGGC
	W96A rev	GCCTTTGTAAGGAACAATCGCCGCATAACCTAAATTTTTGTCG
	Y110A for	GCACAGTTTCAGCTTGGT**GCT**AAAGGATACATCCAGTTAG
	Y110A rev	CTAACTGGATGTATCCTTTAGCACCAAGCTGAAACTGTGC
	K111A for	GCACAGTTTCAGCTTGGTTAT**GCA**GGATACATCCAGTTAGC
	K111A rev	GCTAACTGGATGTATCCTGCATAACCAAGCTGAAACTGTGC
	H185A for	CTCGTAAAGAAATAGAAGCG**GCT**AAACAGAAATTTAGTAAATCGG
	H185A rev	CCGATTTACTAAATTTCTGTTTAGCCGCTTCTATTTCTTTACGAG
	K206A for	CTACGATGCAATGGCTAAA**GCA**ACTGTTCTTAGAAACATGTTAAG
	K206A rev	CTTAACATGTTTCTAAGAACAGTTGCTTTAGCCATTGCATCGTAG
	K206E for	CTACGATGCAATGGCTAAA**GAA**ACTGTTCTTAGAAACATGTTAAG
	K206E rev	CTTAACATGTTTCTAAGAACAGTTTCTTTAGCCATTGCATCGTAG
	R210A for	GGCTAAAAAAACTGTTCTT**GCA**AACATGTTAAGCAAGTGGGGG
	R210A rev	CCCCCACTTGCTTAACATGTTTGCAAGAACAGTTTTTTTAGCC
	R210E for	GGCTAAAAAAACTGTTCTT**GAA**AACATGTTAAGCAAGTGGGGG
	R210E rev	CCCCCACTTGCTTAACATGTTTTCAAGAACAGTTTTTTTAGCC
	N211A for	GGCTAAAAAAACTGTTCTTAGA**GCC**ATGTTAAGCAAGTGGG
	N211A rev	CCCACTTGCTTAACATGGCTCTAAGAACAGTTTTTTTAGCC
	K215A for	CTTAGAAACATGTTAAGC**GCG**TGGGGGATTTTATCCATTGATATG
	K215A rev	CATATCAATGGATAAAATCCCCCACGCGCTTAACATGTTTCTAAG
	K215E for	GAAACATGTTAAGC**GAG**TGGGGGATTTTATCCATTGATATG
	K215E rev	CATATCAATGGATAAAATCCCCCACTCGCTTAACATGTTTC
	K215A/K206A for	CTTAGAAACATGTTAAGC**GCG**TGGGGGATTTTATCCATTGATATG
	K215A/K206A rev	CATATCAATGGATAAAATCCCCCACGCGCTTAACATGTTTCTAAG
	R210A/K206A for	GGCTAAA*GCA*ACTGTTCTT**GCA**AACATGTTAAGCAAGTGGG
	R210A/K206A rev	CCCACTTGCTTAACATGTTTGCAAGAACAGTTGCTTTAGCC
	K111A/K215A for	GCACAGTTTCAGCTTGGTTAT**GCA**GGATACATCCAGTTAGC
	K111A/K215A rev	GCTAACTGGATGTATCCTGCATAACCAAGCTGAAACTGTGC
	R210A/K215A for	GCTAAAAAAACTGTTCTT**GCA**AACATGTTAAGC*GCG*TGGGG
	R210A/K215A rev	CCCCACGCGCTTAACATGTTTGCAAGAACAGTTTTTTTAGC
mutagenesis-outer strand	K101A for	GCGTGGATTGTTCCTTAC**GCA**GGCAGAGCACAGTTTCAG
	K101A rev	GCGTGGATTGTTCCTTACGCAGGCAGAGCACAGTTTCAG
	K101E for	GCGTGGATTGTTCCTTAC**GAA**GGCAGAGCACAGTTTCAG
	K101E rev	CTGAAACTGTGCTCTGCCTTCGTAAGGAACAATCCACGC
	K191A for	CGCATAAACAGAAATTTAGT**GCA**TCGGATTTTGGATGGAAAAAAG
	K191A rev	CTTTTTTCCATCCAAAATCCGATGCACTAAATTTCTGTTTATGCG

	K191E for	CATAAACAGAAATTTAGT**GAA**TCGGATTTTGGATGGAAAAAAG
	K191E rev	CTTTTTTCCATCCAAAATCCGATTCACTAAATTTCTGTTTATG
	F194A for	CAGAAATTTAGTAAATCGGAT**GCT**GGATGGAAAAAAGACTACGAT
	F194A rev	ATCGTAGTCTTTTTTCCATCCAGCATCCGATTTACTAAATTTCTG
	F194E for	CAGAAATTTAGTAAATCGGAT**GAG**GGATGGAAAAAAGACTACG
	F194E rev	CGTAGTCTTTTTTCCATCCCTCATCCGATTTACTAAATTTCTG
	K191A/F194A for	CGCATAAACAGAAATTTAGT**GCA**TCGGAT*GCT*GGATGGAAAAAAG
	K191A/F194A rev	CTTTTTTCCATCCAGCATCCGATGCACTAAATTTCTGTTTATGCG
**Mutagenesis – β1-β2 hairpin wedge**	V98A for	GGTTATGCGTGGATT**GCT**CCTTACAAAGGCAGAGC
	V98A rev	GCTCTGCCTTTGTAAGGAGCAATCCACGCATAACC
	V98W for	GGTTATGCGTGGATT**TGG**CCTTACAAAGGCAGAGCACAGTTTC
	V98W rev	GAAACTGTGCTCTGCCTTTGTAAGGCCAAATCCACGCATAACC
	Y100A for	GCGTGGATTGTTCCT**GCC**AAAGGCAGAGCACAGTTTCAG
	Y100A rev	CTGAAACTGTGCTCTGCCTTTGGCAGGAACAATCCACGC
	Y100E for	GGTTATGCGTGGATTGTTCCT**GAG**AAAGGCAGAGCACAG
	Y100E rev	CTGTGCTCTGCCTTTCTCAGGAACAATCCACGCATAACC
	Q107A for	GGCAGAGCACAGTTT**GCG**CTTGGTTATAAAGGATACATC
	Q107A rev	GATGTATCCTTTATAACCAAGCGCAAACTGTGCTCTGCC
	Q107H for	CAAAGGCAGAGCACAGTTT**CAC**CTTGGTTATAAAGGATAC
	Q107H rev	GTATCCTTTATAACCAAGGTGAAACTGTGCTCTGCCTTTG
	V98A/Y100A for	GGTTATGCGTGGATT**GCT**CCT*GCC*AAAGGCAGAGC
	V98A/Y100A rev	GCTCTGCCTTTGGCAGGAGCAATCCACGCATAACC
	Q107A/V98A/Y100A for	*GCC*AAAGGCAGAGCA**GCG**TTTCAGCTTGGTTATAAAGG
	Q107A/V98A/Y100A rev	CCTTTATAACCAAGCTGAAACGCTGCTCTGCCTTTGGC
**Mutagenesis – subunit interface**	L118A for	GGATACATCCAGTTAGCA**GCA**CGCACAGGACAATATAAAAGC
	L118A rev	GCTTTTATATTGTCCTGTGCGTGCTGCTAACTGGATGTATCC
	I126A for	CGCACAGGACAATATAAAAGC**GCT**AATGTTATCGAAGTGCGCG
	I126A rev	CGCGCACTTCGATAACATTAGCGCTTTTATATTGTCCTGTGCG
	I126H for	CGCACAGGACAATATAAAAGC**CAT**AATGTTATCGAAGTGCGC
	I126H rev	GCGCACTTCGATAACATTATGGCTTTTATATTGTCCTGTGCG
	F171A for	CTATTTCCAGTTAATTAATGGC**GCT**GAAAAAACGGTCTATTGGAC
	F171A rev	GTCCAATAGACCGTTTTTTCAGCGCCATTAATTAACTGGAAATAG
	W216A for	CTTAGAAACATGTTAAGCAAG**GCG**GGGATTTTATCCATTGATATG
	W216A rev	CATATCAATGGATAAAATCCCCGCCTTGCTTAACATGTTTCTAAG
	W216R for	CTTAGAAACATGTTAAGCAAG**AGG**GGGATTTTATCCATTGATATG
	W216R rev	CATATCAATGGATAAAATCCCCCTCTTGCTTAACATGTTTCTAAG
	I218A for	CATGTTAAGCAAGTGGGGG**GCT**TTATCCATTGATATGCAAACAG
	I218A rev	CTGTTTGCATATCAATGGATAAAGCCCCCCACTTGCTTAACATG
	I218E for	GAAACATGTTAAGCAAG**GAG**GGGATTTTATCCATTGATATGC
	I218E rev	GCATATCAATGGATAAAATCCCCTCCTTGCTTAACATGTTTC
	L118A/F171A for	GGATACATCCAGTTAGCA**GCA**CGCACAGGACAATATAAAAGC
	L118A/F171A rev	GCTTTTATATTGTCCTGTGCGTGCTGCTAACTGGATGTATCC
	K215A/W216A for	CTTAGAAACATGTTAAGC**GCG**TGGGGGATTTTATCCATTGATATG
	K215A/W216A rev	CATATCAATGGATAAAATCCCCCACGCGCTTAACATGTTTCTAAG

[1]All DNA sequences are written in the 5’-3’ direction. For mutagenesis primers, the codon being mutated is indicated in bold with the nucleotide changes underlined. For double and triple mutants, the codon of the first (existing) mutation is underlined and in italics and are named in reverse order of incorporation (the first mutation indicated was the last to be incorporated).

Supplementary Movie 1.

The movie was generated in PyMOL (5). Captions to the movie were inserted using Adobe Premier, with the following transcript:

“This movie shows seven subunits of LiRecT protein bound to 31 bp of DNA (5 bp/monomer).

The DNA is bound in a highly extended and under-wound conformation that appears to be a novel duplex intermediate of DNA annealing.

The complex was prepared by mixing LiRecT with two complementary strands of ssDNA that were added to the protein sequentially.

The yellow strand, which we call “inner” is almost certainly the first strand added. It is bound deep in a groove with its bases exposed for homology recognition.

Several side chains of the protein contact the sugar phosphate backbone of the inner strand, and hold it in an irregular conformation that is periodically kinked.

The orange strand, which we call “outer”, forms fewer contacts with the protein, and is held in place primarily by Watson-Crick base pairs with the inner strand.

While most of the contacts are to the sugar-phosphate backbone of the DNA, a beta-hairpin of the protein wedges into the base pairs to separate them, at every 5 bp step.

Neighboring subunits of LiRecT bind to one another via two different sets of interactions.

The first set, above the DNA binding groove, involves the N-terminal helix bundles, which form a core of hydrophobic interactions surrounded by a few ion pairs.

A second set of interactions, below the DNA binding groove, forms a similar hydrophobic core using the three-stranded beta-sheet and two central alpha helices.

We hypothesize that the N-terminal helix bundles clamp down on the duplex after the second strand is added, to stabilize the complex and consolidate annealing.”

Supplementary References

1. Caldwell, B. J. *et al.*, Oligomeric complexes formed by Redβ single strand annealing protein in its different DNA bound states. *Nucleic Acids Res* **49**, 3441–3460 (2021).
2. Punjani, A., Rubinstein, J. L., Fleet, D. J. & Brubaker, M. A. cryoSPARC: algorithms for rapid unsupervised cryo-EM structure determination. *Nature Methods* 14, 290–296 (2017).
3. Pettersen E. F. *et al.* UCSF ChimeraX: Structure visualization for researchers, educators, and developers. *Protein Sci.* **30**, 70-82 (2021).
4. Emsley, P., Lohkamp, B., Scott, W. G., Cowtan, K. Features and development of Coot. *Acta Crystallogr.* **D66**, 486-501 (2010).
5. The PyMOL Molecular Graphics System, Version 2.5, Schrödinger, LLC.
6. Holm, L. Dali server: structural unification of protein families. *Nucleic Acids Res*. **50**, W210-W215 (2022).
7. Baek, M. *et al.* Accurate prediction of protein structures and interactions using a three-track neural network. *Science* **373**, 871–876 (2021).
8. Sievers, F., *et al.* Fast, scalable generation of high-quality protein multiple sequence alignments using Clustal Omega. *Mol. Syst. Biol.* **7**, 539 (2011).
9. Lu, X.-J. & Olson, W. K. 3DNA: a versatile, integrated software system for the analysis, rebuilding and visualization of three-dimensional nucleic-acid structures. *Nat. Protoc*. **3**, 1213–1227 (2008).
10. Saotome, M. *et al.* Structural basis of homology-directed DNA repair mediated by RAD52. *iScience* **3**, 50–62 (2018).
