# Supplementary material for: Structure of a RecT/Redβ family recombinase in complex with a duplex intermediate of DNA annealing
Source: Nat Commun. 2022 Dec 21;13:7855. doi: 10.1038/s41467-022-35572-z (PMC9772228; doi:10.1038/s41467-022-35572-z)
Supplement: Supplementary file 3 — Description of Additional Supplementary Files [file 41467_2022_35572_MOESM3_ESM.pdf]

Description of Additional Supplementary Files

File name: Supplementary Movie 1
Description: PyMOL Movie of the LiRecT protein in complex with a novel duplex intermediate of DNA annealing. The movie was generated in PyMOL and captions to the movie were inserted using Adobe Premier.
